# Enlarging the Stokes Shift by Weakening the *π*‐Conjugation of Cyanines for High Signal‐to‐Noise Ratiometric Imaging

**DOI:** 10.1002/advs.202205080

**Published:** 2022-11-24

**Authors:** Yongkang Yue, Tingting Zhao, Zhou Xu, Weijie Chi, Xiaojun Chai, Jiahong Ai, Jiawei Zhang, Fangjun Huo, Robert M. Strongin, Caixia Yin

**Affiliations:** ^1^ Key Laboratory of Chemical Biology and Molecular Engineering of Ministry of Education Institute of Molecular Science Shanxi University Taiyuan 030006 China; ^2^ School of Science Hainan University Renmin Road 58 Haikou 570228 China; ^3^ Second People's Hospital in the City of Linfen Linfen 041099 China; ^4^ Research Institute of Applied Chemistry Shanxi University Taiyuan 030006 China; ^5^ Department of Chemistry Portland State University Portland OR 97201 USA

**Keywords:** cyanine, imaging, nano material, signal‐to‐noise ratio, Stokes shift

## Abstract

The signal‐to‐noise ratio (SNR) is one of the key features of a fluorescent probe and one that often defines its potential utility for in vivo labeling and analyte detection applications. Here, it is reported that introducing a pyridine group into traditional cyanine‐7 dyes in an asymmetric manner provides a series of tunable NIR fluorescent dyes (Cy‐Mu‐7) characterized by enhanced Stokes shifts (≈230 nm) compared to the parent cyanine 7 dye (<25 nm). The observed Stokes shift increase is ascribed to symmetry breaking of the Cy‐Mu‐7 core and a reduction in the extent of conjugation. The fluorescence signals of the Cy‐Mu‐7 dyes are enhanced upon confinement within the hydrophobic cavity of albumin or via spontaneous encapsulation within micelles in aqueous media. Utilizing the Cy‐Mu‐7, ultra‐fast in vivo kidney labeling in mice is realized, and it is found that the liver injury will aggravate the burden of kidney by monitoring the fluorescence intensity ratio of kidney to liver. In addition, Cy‐Mu‐7 could be used as efficient chemiluminescence resonance energy transfer acceptor for the reaction between H_2_O_2_ and bisoxalate. The potential utility of Cy‐Mu‐7 is illustrated via direct monitoring fluctuations in endogenous H_2_O_2_ levels in a mouse model to mimic emergency room trauma.

## Graphical abstract

In this work, it is shown that asymmetric introduction of a pyridine group into a cyanine‐7 core gives rise to a series of tunable NIR fluorescent dyes, Cy‐Mu‐7, characterized by large Stokes shifts (>200 nm) and high SNRs when used for in vivo imaging.

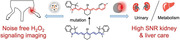

## 1 Introduction

Organic fluorescent dyes are among the most widely used labeling tools. They have enabled unprecedented insights into both cellular and in vivo processes and are appealing because of the relatively noninvasive nature of emission‐based optical analyses.^[^ ^1^, ^2^ ^]^ However, the performance of many common organic fluorescent dyes is restricted by their low signal‐to‐noise ratio (SNR). This is particularly problematic in the context of in vivo imaging applications.^[^ ^3^, ^4^, ^5^ ^]^ Not surprisingly, therefore, in recent years scientists have worked to enhance the inherent signal intensity, reduce the noise or optimize both parameters to improve the SNR.^[^ ^6^ ^]^

Molecular features that could improve the SNR include 1) increasing the fluorescence emission wavelength; 2) enhancing the Stokes shift; and 3) targeting the fluorescence or chemiluminescence.^[^ ^7^ ^]^ The benefits of the first approach is that fluorescence emission in the NIR region would not only avoid the noise caused by tissue autofluorescence, but also allow for improved signal readout as the result of better tissue penetration and lower light scattering.^[^ ^8^ ^]^ Since they were reported by Williams in 1856, cyanine dyes, especially heptamethine cyanines (Cy‐7), have been used extensively as NIR photosensitizers and fluorescent probes.^[^ ^9^, ^10^, ^11^, ^12^ ^]^ The second strategy for increasing the SNR, namely improving the Stokes shift, is particularly germane to Cy‐7 dyes, which typically feature narrow Stokes shifts, generally less than 25 nm (Figures S1 and S2, Supporting Information). This small shift results in so‐called crosstalk between the excitation and emission bands and results in a diminished SNR relative to what would otherwise be expected.^[^ ^10^, ^13^, ^14^ ^]^ In fact, in the case of several recently reported cyanine‐based NIR‐II dyes, their narrow Stokes shifts was recognized as being one of the key parameters that limited their utility in bio‐imaging applications.^[^ ^11^ ^]^ The third approach to improving the SNR has been widely exploited to construct bio‐labeling tools. However, further benefits might be expected if the Stokes shift could be enhanced, especially in the case of cyanine‐based NIR‐II dyes. Focused on the rational molecular design of satisfactory in vivo imaging dyes, we hope to improve the restrictions such as narrow Stokes shifts and always‐ON fluorescence signals of Cy‐7 while preserve their NIR fluorescence emission feature. We found that asymmetrical introduction of a pyridine group to the symmetric Cy‐7 dyes would reduce the conjugate effect and lead to a >200 nm increase in the Stokes shift. For convenience, we refer to these new derivatives as Cy‐Mu‐7 dyes.

The Cy‐Mu‐7 dyes bind spontaneously to the low‐polarity hydrophobic cavity of albumin (domain IIIb) when mixed in aqueous media. This binding is likely driven by the hydrophobic effect, restricts the rotation of the pyridine moiety, and enhances the fluorescence signal (6.4‐fold enhancement comparing with the Cy‐7). Increases in emission intensity were also seen when the Cy‐Mu‐7 dyes were encapsulated in micelles such as Pluronic F‐127, a commercial copolymer surfactant. Cy‐Mu‐7 was found to bind to serum albumin after IV injection. Within 1 min post‐injection, the kidneys were found to light up (luminesce) and with a high SNR compared with ICG or indole‐Cy‐7. This allowed Cy‐Mu‐7 to be used to image in vivo in nephrotoxic drug‐induced albuminuria, one of the biomarkers of acute kidney injury (AKI), at an early stage of AKI.^[^ ^15^, ^16^ ^]^ Cy‐Mu‐7 was also encapsulated in Pluronic F‐127 along with bis[3,4,6‐trichloro‐2‐(pentyl‐oxycarbonyl)phenyl]oxalate (CPPO), a species that produces chemiluminescence in the presence of hydrogen peroxide (H_2_O_2_). The nanoprobe prepared in this way allows visualization of the transient production and fluctuation of endogenous H_2_O_2_ as the result of chemiluminescence resonance energy transfer (CRET) between the CPPO‐derived 1,2‐dioxetanedione and the Cy‐Mu‐7 dye.^[^ ^17^, ^18^ ^]^ This system could be applied to a murine model for emergency room trauma.

## 2 Results and Discussion

### 2.1 Synthesis of the Asymmetrical Cy‐Mu‐7 Dyes

Detailed synthetic protocols and characterizations are presented in Supporting Information. Typically, the *N*‐methyl‐indole derivative (Cy‐Mu‐7) could be readily synthesized via two steps asymmetrical insertion of the *N*‐methyl‐indole moiety and pyridine moiety successively. For the *N*‐propionyloxy‐indole derivative (Cy‐Mu‐7‐COOH), the presence of hyamine could catalyze ester condensation reaction in EtOH with the formation of an *N*‐propionic ether derivative (Cy‐Mu‐7‐ester). So, we used acetonitrile and methylbenzene as the solvents in the two steps Cy‐Mu‐7‐COOH synthesis, respectively. Based on a similar synthetic protocol, the benzindole‐modified derivative could also be obtained (Cy‐Mu‐7‐benzene).

### 2.2 Spectral Properties of Cy‐Mu‐7 Dyes

**Figure 1:**
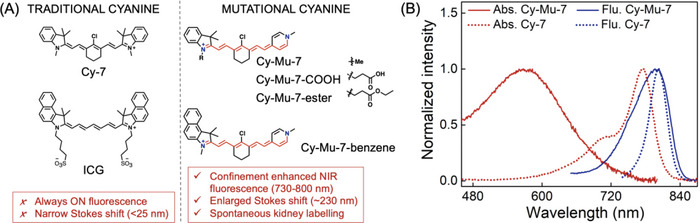
A) Structures of traditional Cy‐7 dyes and the asymmetrical Cy‐Mu‐7 dyes and B) their representative photophysical properties in PBS.

Cy‐Mu‐7 features typical absorption band centered at 570 nm in PBS with a molar extinction coefficient (*ε*) of 11 760 m ^−1^ cm^−1^ (**Figure** **1** and **Table** **1**). Compared with the traditional symmetric Cy‐7 (775 nm, *ε* = 125 510 m ^−1^ cm^−1^), the maximum absorption peak of Cy‐Mu‐7 was reduced by ≈200 nm. Even though with distinct absorption bands, Cy‐Mu‐7 and Cy‐7 displayed similar fluorescence emission spectra centered at ≈800 nm in PBS. These spectral results indicated that Cy‐Mu‐7 reserved the NIR fluorescence emission of heptamethine cyanine while featured >200 nm Stokes shift.

**Table 1:** Photophysical properties of Cy‐Mu‐7 dyes, Indole‐Cy‐7, and ICG with or without HSA. The absolute fluorescence quantum yields of the dyes were determined in PBS

Dyes		Abs. [nm]	Fl. [nm]	*ε* [10^5^ m ^−1^ cm^−1^]	*Φ* [%]	Brightness *εΦ* [M^−1^ cm^−1^]	Stokes shift [nm]
Cy‐Mu‐7	–	570	800	0.1176	1.26	148.176	230
	+ HSA	600	755	0.4869	2.59	1261.071	155
Cy‐Mu‐7‐COOH	–	574	800	0.1371	0.62	85.002	226
	+ HSA	580	775	0.1251	0.99	123.849	195
Cy‐Mu‐7‐ester	–	565	790	0.2074	0.33	68.442	225
	+ HSA	573	736	0.2331	1.68	391.608	163
Cy‐Mu‐7‐benzene	–	550	804	0.1471	0.56	82.376	254
	+ HSA	610	775	0.2695	3.94	1061.83	165
Indole‐Cy‐7	–	775	803	1.2551	2.77	3476.627	28
	+ HSA	797	815	1.2651	4.6	5819.46	18
ICG	–	777	804	0.6706	1.53	1026.018	27
	+ HSA	796	816	1.5629	2.94	4594.926	20

**Figure 2:**
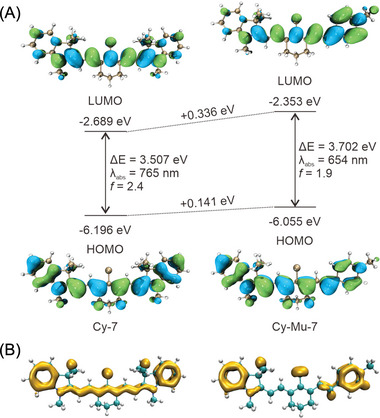
Computational results of Cy‐7 and Cy‐Mu‐7 in water. A) The calculated distributions, energy levels (*E*_HOMO_ and *E*_LUMO_) of HOMO and LUMO, energy gaps (Δ*E*, Δ*E* = *E*_LUMO_ – *E*_HOMO_), maximum absorption wavelength (*λ*_abs_), and oscillator strength (*f*) of Cy‐7 and Cy‐Mu‐7. B) Iso‐surface map of LOL‐*π* of Cy‐7 and Cy‐Mu‐7 with iso‐value of 0.4 at M06‐2X/6‐31G(d) level.

Density functional theory (DFT) and time‐dependent DFT (TD‐DFT) were then employed to understand the structural and electronic properties of Cy‐7 and Cy‐Mu‐7 in the Gaussian 16 A code.^[^ ^19^ ^]^ As shown in **Figure** **2**A, both of the highest occupied molecular orbital (HOMO) and the lowest unoccupied molecular orbital (LUMO) of Cy‐7 and Cy‐Mu‐7 are located in the whole molecules. Compared with the highly symmetrical electron density distributions of Cy‐7 during the HOMO → LUMO transition, the excitation process of Cy‐Mu‐7 induced distinct electron density changes from the indole moiety to the pyridine moiety. The calculated centroid distances between the HOMO and LUMO of Cy‐7 and Cy‐Mu‐7 were 0.37 and 5.40 Å, respectively, which indicated a larger Stokes shift of Cy‐Mu‐7 over Cy‐7.^[^ ^20^ ^]^ The HOMO and LUMO levels of Cy‐7 and Cy‐Mu‐7 also reflected the spectral differences. The asymmetrical introduction of a pyridine moiety elevated the LUMO energy level of the cyanine significantly (Δ*E*_LUMO_ = 0.366 eV). As a result, Cy‐Mu‐7 (3.702 eV) exhibits a larger energy gap than Cy‐7 (3.507 eV), which leads to a significantly hypochromatic shift of the maximum absorption peak of Cy‐Mu‐7 compared to Cy‐7. The oscillator strength (*f*), which was positively correlated to the *ε* of the dyes, of Cy‐Mu‐7 (*f* = 1.9) was also reduced by ≈26% compared to Cy‐7 (*f* = 2.4).^[^ ^21^ ^]^ All of these computational results were in good agreement with the experimental spectral data.

The changes in maximum absorption peak and oscillator strength in Cy‐Mu‐7 can be rationalized by *π* electron distribution, which was characterized by Localized Orbital Locator (LOL‐*π*).^[^ ^22^, ^23^ ^]^ We showed that the LOL‐*π* of Cy‐Mu‐7 exhibits a narrower distribution compared with Cy‐7, which results in a reduction of the conjugate effect (Figure 2B). In other words, the hypochromatic shift of the maximum absorption peak and reduction of oscillator strength mainly stems from the weak conjugate effect of Cy‐Mu‐7. From the point of view of molecular structure, the symmetry breaking of Cy‐Mu‐7 caused the reduction of the conjugate effect.

The molecular design strategy to construct heptamethine cyanine dyes with large Stokes shift could be verified by the spectral properties of Cy‐Mu‐7 derivatives. All of these dyes featured similar UV–vis absorption and NIR fluorescence emission properties in PBS (Figure S3, Supporting Information and Table 1). The corresponding Stokes shifts varied from 225 to 254 nm. Cy‐Mu‐7‐COOH and Cy‐Mu‐7‐ester supported the feasibility of these dyes for diverse biological applications upon further modification.

**Figure 3:**
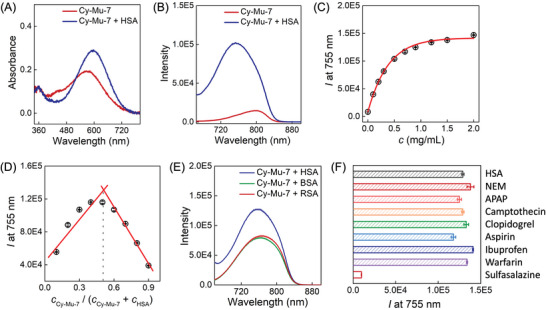
Optical properties of Cy‐Mu‐7 in the presence of human serum albumin (HSA). A) UV–vis absorption changes upon addition of 1 mg mL^−1^ HSA into 10 µm Cy‐Mu‐7 containing PBS system. B) The corresponding fluorescence spectra changes of (A). C) HSA concentration dependent fluorescence intensity changes of 10 µm Cy‐Mu‐7 containing PBS system. D) Fluorescence intensity (*I* = 755 nm) analysis of the mixture containing Cy‐Mu‐7 and HSA with different ratios, the overall concentration of the mixture was 20 µm. E) Fluorescence responses of 10 µm Cy‐Mu‐7 toward 1 mg mL^−1^ HSA, rat serum albumin (RSA) or bovine serum albumin (BSA). F) Fluorescence intensity changes of 10 µm Cy‐Mu‐7 in the presence of 1 mg mL^−1^ HSA pre‐incubated with 400 µm various compounds including ibuprofen, warfarin, acetaminophen (APAP), aspirin, sulfasalazine, camptothecin, clopidogrel, and *N*‐ethylmaleimide (NEM), respectively. *λ*_ex_ = 610 nm; slit 6/6 nm. Error bars represent standard deviations obtained from three independent experiments.

Since the NIR fluorescence dyes are preferred in vivo imaging tools, we evaluated the spectral responses of Cy‐Mu‐7 dyes toward albumin, the most abundant protein in animal plasma, to support the potential in vivo imaging applications. HSA as a typical albumin was primarily evaluated in the following experiments. All of these Cy‐Mu‐7 dyes could bind HSA with similar spectral changes. Typically, the presence of HSA induced bathochromic shift of the absorption wavelength and hypochromatic shift of the fluorescence emission wavelength in PBS. At the same time, the increased *ε* and fluorescence quantum yield (*Φ*) after HSA binding brought significant fluorescence brightness enhancement of the dyes containing PBS solution. For the *N*‐methyl‐indole Cy‐Mu‐7, HSA binding induced 11‐fold fluorescence intensity enhancement at 755 nm (**Figure** **3**B). The corresponding Stokes shifts changed from 230 nm (Cy‐Mu‐7) to 155 nm (Cy‐Mu‐7‐HSA). The fluorescence intensity of HSA added Cy‐Mu‐7 system was HSA concentration‐dependent with a *K*_a_ (association constant) of 1.9 × 10^5^ m ^−1^ (Figure 3C and Figure S4, Supporting Information). The binding molar ratio of Cy‐Mu‐7 and HSA was measured to be 1: 1 based on the equimolar method (Figure 3D).^[^ ^24^ ^]^ Besides, RSA and BSA could also bind to Cy‐Mu‐7 with similar fluorescence responses in PBS, the corresponding fluorescence intensities were weaker compared with the HSA addition system under the same condition (Figure 3E). In the case of Cy‐7, however, HSA binding induced negligible fluorescence brightness changes in aqueous solution. The potential in vivo imaging properties improvement could be evaluated by covering muscle tissue upon the solutions. Figure S5 (Supporting Information) displays the fluorescence imaging results of 10 µm Cy‐Mu‐7 and Cy‐7 with/without HSA upon 0.5 mm pork covering. 6.4‐fold fluorescence signal enhancement was presented for the Cy‐Mu‐7 over Cy‐7 case. Thus, the new Cy‐Mu‐7 dyes are promising as activable NIR fluorescence probes for albumin related physiological processes and diseases diagnosis.

The binding mode of Cy‐Mu‐7 and HSA was then analyzed by SDS‐PAGE gel and no fluorescence signal appeared in the HSA band after electrophoresis which meant the non‐covalent binding mode (Figure S6, Supporting Information). We then performed competitive inhibition experiments upon the addition of the reported HSA non‐covalent binding compounds with different binding domains to determine its non‐covalent binding site. As shown in Figure 3F (detailed in Figure S7, Supporting Information), upon pre‐incubation of compounds including ibuprofen, warfarin, APAP, aspirin, NEM, sulfasalazine, camptothecin, and clopidogrel with HSA, respectively, only sulfasalazine exhibited the further combination of Cy‐Mu‐7. Sulfasalazine with high concentration was reported to bind to HSA with a molar ratio of 3:1.^[^ ^25^ ^]^ The three binding sites were Sudlow‐site I, domain IIIb and the interface between domains IIIa and IIIb of HSA with increasing dissociation constants (Figure S8, Supporting Information). However, considering the high affinity of warfarin in the center of Sudlow‐site I, domain IIIb might be the preferred binding site for Cy‐Mu‐7.^[^ ^26^ ^]^ Supported by these results, molecular docking calculations were conducted to verify the binding mode of Cy‐Mu‐7 or Cy‐Mu‐7‐ester with the ligand‐removed crystal structure of HSA (PDB ID: 6R7S).^[^ ^25^, ^27^ ^]^ 23 and 142 distinct conformational clusters were found for Cy‐Mu‐7 and Cy‐Mu‐7‐ester based docking out of 2000 runs, respectively. Both of them presented domain IIIb as the energetically favorable binding domain which is overlapped exactly with the reported binding site of sulfasalazine (Figure S9, Supporting Information). Typically, the hydrophobic alkene moiety of Cy‐Mu‐7 intercalated with the strong hydrophobic area of domain IIIb formed by Phe‐507, Phe‐509, and Phe‐551 (Figure S9A, Supporting Information). The pyridine moiety was embedded in the cavity of the domain while the *N*‐methyl‐indole moiety as the rivet was stuck over the domain which permitted the further modification of Cy‐Mu‐7. In the following solvents‐dependent spectroscopic studies, we noticed that the lowered polarity would induce bathochromic‐shift of the absorption wavelength of Cy‐Mu‐7 and enhanced viscosity would permit the blue‐shift of the fluorescence signal (Figure S10, Supporting Information). Thus, the low‐polarity hydrophobic environment which further restricted the motion of pyridine moiety induced the fluorescence enhancement. In addition, Cy‐Mu‐7 did not respond to biological amino acids and the activated fluorescence system of Cy‐Mu‐7‐HSA was inert in aqueous solution even in the presence of reactive oxygen species (Figure S11, Supporting Information). These results provide support for the notion that Cy‐Mu‐7 warrants consideration as a probe for albumin‐related in vivo imaging.

### 2.3 In Vivo and In Vitro Diagnostic Imaging by Cy‐Mu‐7

In order to perform biological imaging experiments, CCK‐8 assay was then used to evaluate the cytotoxicity of Cy‐Mu‐7 in HeLa cells. After incubation for 12 h, 5 µm Cy‐Mu‐7 did not induce significant cell death (Figure S12, Supporting Information). So, 5 µm Cy‐Mu‐7 was used to incubate cells in the following cell imaging experiments. Compared with the cells incubated with Cy‐Mu‐7 containing PBS directly, HSA loaded cells displayed significant fluorescence enhancement in the NIR channel (Figure S13, Supporting Information). The results demonstrated that Cy‐Mu‐7 was membrane permeable and could bind to HSA in situ in living HeLa cells with turn‐on fluorescence signal.

**Figure 4:**
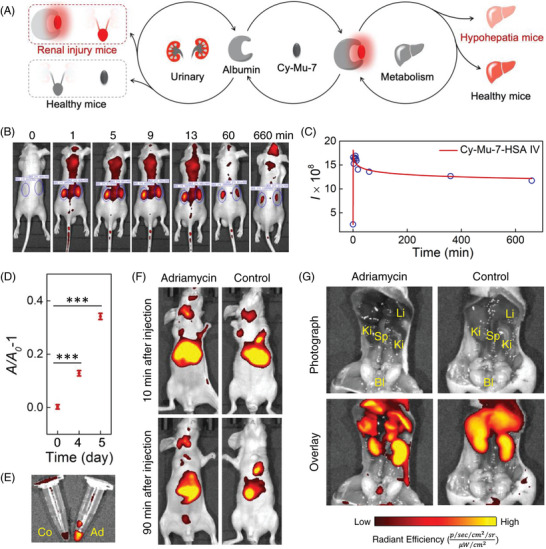
Metabolism of Cy‐Mu‐7 in mice upon IV injection and AKI in vivo imaging. A) The illustration of diseases prediction based on in vivo fluorescence distribution. B) Time‐dependent in vivo fluorescence imaging of mouse IV injected with 100 µL PBS containing 160 µm Cy‐Mu‐7 and 10 mg mL^−1^ HSA and C) the corresponding average fluorescence intensities of the ROI in (B). D) Analysis of protein concentration in urine by a BCA Protein Assay Kit. 10 mg kg^−1^ Adriamycin (water solution, 100 µL/20 g body weight) was administrated upon IV injection. E) Fluorescence images of urine samples collected from the 5‐day‐Adriamycin administrated mice and healthy mice within 2 h after Cy‐Mu‐7‐COOH‐HSA (PBS solution containing 160 µm Cy‐Mu‐7‐COOH and 10 mg mL^−1^ HSA, 100 µL/20 g body weight) injection, respectively. F) Time‐dependent in vivo fluorescence imaging of 5‐day‐Adriamycin administrated and healthy mice after IV injection of 100 µL PBS solution containing 160 µm Cy‐Mu‐7‐COOH and 10 mg mL^−1^ HSA. G) Ex vivo fluorescence images of abdominal cavity of 5‐day‐Adriamycin administrated and healthy mice at 90 min after IV injection of 100 µL PBS solution containing 160 µm Cy‐Mu‐7‐COOH and 10 mg mL^−1^ HSA. Error bars represent standard deviations obtained from three independent experiments. Ki, kidney; Li, liver; Bl, bladder; Sp, spleen.

Exogenous substances are generally cleared through the synergistic effect of liver metabolism and renal excretion after IV injection into the blood circulation system.^[^ ^28^ ^]^ The glomerular filtration membrane of the kidney is constituted by negatively charged layers to prevent the filtration of natural proteins but permit the clearance of positively charged molecules owing to electrostatic interactions.^[^ ^29^, ^30^ ^]^ Thus, we assumed that the positively charged Cy‐Mu‐7 might label kidney efficiently after binding to albumin spontaneously with NIR fluorescence emission (**Figure** **4**A). The Cy5.5 imaging channel with the excitation at 605 nm was used in the following in vivo imaging experiments. As shown in Figure S14 (Supporting Information), IV injection of 160 µm Cy‐Mu‐7 in PBS or PBS solution containing 160 µm Cy‐Mu‐7 and 10 mg mL^−1^ HSA induced distinct fluorescence signal enhancement in the kidneys of mice which demonstrated our assumption that Cy‐Mu‐7 could be used for kidney labeling in vivo. Consistent with the fluorescence spectral data, Cy‐Mu‐7‐HSA featured stronger fluorescence signal in vivo. So, in the following in vivo experiments, pre‐bound Cy‐Mu‐7‐HSA was used to ensure higher signal to noise ratio. Besides, Cy‐Mu‐7‐ester and Cy‐Mu‐7‐COOH could also label kidney instantaneously after IV injection (Figure S15, Supporting Information). In a following time‐dependent in vivo imaging experiment, we noticed that the kidneys of mice could be lightened within 1 min with fivefold fluorescence signal enhancement after IV injection (Figure 4B,C) which is as faster as the present reported renal‐clearable fluorescence dyes.^[^ ^31^ ^]^ For traditional Cy‐7 dyes, however, after IV injection of Indole‐Cy‐7 or ICG at the same dose as Cy‐Mu‐7, the mice showed systemic fluorescence enhancement, especially in the liver, which masked the potential renal labeling of these dyes (Figure S16, Supporting Information). The results further highlighted the important structural modification of Cy‐Mu‐7 dyes for better in vivo imaging applications.

The kidneys excrete metabolic wastes produced in the body with the urine being subject to glomerular blood filtration and renal tubular secretion. In the early stage of AKI diseases such as acute glomerular inflammation and acute interstitial nephritis, the urine protein concentration increases significantly, resulting in albuminuria.^[^ ^15^, ^32^ ^]^ Cy‐Mu‐7 dyes displayed weak fluorescence in aqueous solution while strong NIR fluorescence emission after binding to albumin. So, we considered it like that albuminuria in an AKI model might lead to the bladder lightening upon after Cy‐Mu‐7 injection. Adriamycin induced nephropathy was reported to be an experimental analogue of human minimal lesion nephrotic syndrome.^[^ ^33^, ^34^ ^]^ BALB/c mice developed nephrotic syndrome in the first two weeks and aggravated in the later period after IV administration of 10 mg kg^−1^ Adriamycin.^[^ ^35^ ^]^ We collected daily urine samples of Adriamycin injected mice and analyzed the protein concentrations with a BCA Protein Assay Kit. As shown in Figure 4D, the protein concentration increased significantly within 5 d after Adriamycin administration which was consistent with the reported symptom.^[^ ^35^ ^]^ We then injected the complex of Cy‐Mu‐7‐COOH‐HSA intravenously to Adriamycin injected (5 d after administration) model mice and healthy mice, respectively. The time‐dependent fluorescence signal changes of the kidneys did not display a significant difference between the Adriamycin and control groups (Figure S17, Supporting Information). In the ventral images, however, a distinct fluorescence signal appeared in the bladder of the Adriamycin group 90 min after Cy‐Mu‐7‐COOH‐HSA injection (Figure 4F). The in vivo fluorescence monitoring results were further confirmed by the fluorescence images of urine samples collected within 2 h after Cy‐Mu‐7‐COOH‐HSA injection. Adriamycin group resulted in stronger uric fluorescence compared with the control group (Figure 4E). The corresponding fluorescence images of the abdominal cavity of mice further illustrated the distribution of fluorescence signals in both of the Adriamycin and control groups (Figure 4G). In the subsequent H&E staining of kidney tissue resected from the corresponding Adriamycin administrated mice, no significant histological lesion was found compared with the control group (Figure S18, Supporting Information). The present results demonstrated that the new NIR dyes could be used as a sensitive tool for early‐stage of AKI imaging in vivo.

**Figure 5:**
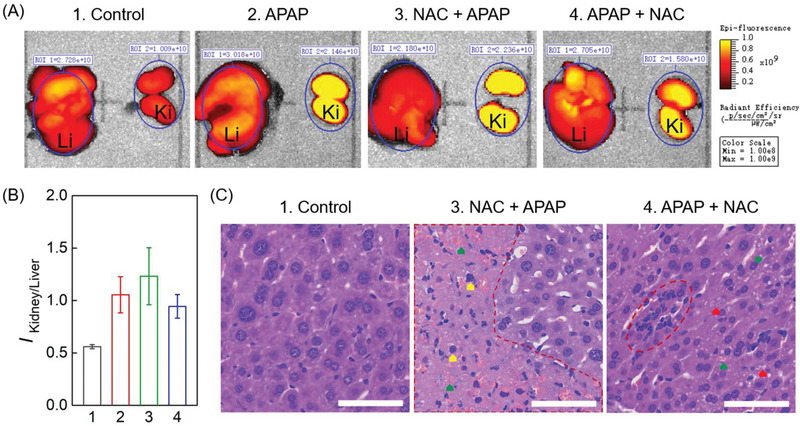
Fluorescence intensity ratios of kidney‐to‐liver for drug induced liver injury imaging. A) Fluorescence images of kidney and liver organs harvested from mice, which were IP injected with 1. saline, 2. 24 mg APAP, 3. 16 mg NAC and 24 mg APAP 1 h after NAC injection, 4. 24 mg APAP and 16 mg NAC 1 h after APAP injection and further IV injected with PBS solution containing 160 µm Cy‐Mu‐7 and 10 mg mL^−1^ HSA 24 h after APAP injection, 5 h after the dye injection. B) Corresponding K/L ratios of (A). C) H&E staining of the corresponding liver tissues of (A). 6‐week‐old male ICR mice were used in these experiments. Error bars represent standard deviations obtained from three independent experiments. Ki, kidney; Li, liver. Scale bar, 60 µm.

The IV injected Cy‐Mu‐7 metabolized through both kidney and liver. Interestingly, we found that the fluorescence intensity ratios of kidney‐to‐liver (K/L ratio) were age‐dependent (Figure S19, Supporting Information). Considering that age was reported to independently predict hepatocellular death or transplantation and associated with an increased risk of death, we constructed acute liver injury mice model upon IP injection of over dosage APAP (600 mg kg^−1^, sodium carboxymethyl cellulose suspension) to evaluate the metabolic proportion modulation.^[^ ^36^, ^37^ ^]^ Comparing with the control group, APAP group displayed a twofold K/L ratio of the corresponding fluorescence intensities (**Figure** **5**A‐1, A‐2,B). This result might be caused by the drug‐induced liver failure which decreased the clearance of exogenous substances through the reticuloendothelial system and, thus, increased their renal clearance proportion. *N*‐acetylcysteine (NAC) has a significant therapeutic effect in the treatment of APAP‐induced acute liver injury.^[^ ^38^, ^39^ ^]^ In our experiments, we tested the dosing order induced difference in the therapeutic effect of NAC. As shown in Figure 5A‐3, IP injection of 400 mg kg^−1^ NAC 1 h before APAP administration (NAC‐APAP group) did not reverse the K/L ratio compared with the APAP group. However, NAC injected 1 h after APAP administration (APAP‐NAC group) could induce a distinct K/L ratio decrease compared with the APAP or NAC‐APAP group (Figure 5A‐4,BB). The organs’ fluorescence imaging results were further verified by histopathological analysis (Figure 5C and Figure S20, Supporting Information). H&E staining of the corresponding liver tissues illustrated that significant tissue necrosis appeared (the red‐dotted outline) in the NAC‐APAP group. Besides, distinct focal hemorrhage (green arrow) and nuclear pyknosis (yellow arrow) could be found in the slice. The condition was greatly improved in the APAP‐NAC group which displayed small scale inflammatory infiltrate (the red‐dotted outline) and red blood cells extravasate (green arrow). The dosing order dependent therapeutic effect difference might be caused by the efficient pharmacokinetics of NAC upon IP administration.^[^ ^38^, ^40^ ^]^ The corresponding liver function decline induced renal clearance pressure exacerbation of exogenous substances might explain the undigested fact that non‐alcoholic fatty liver diseases are independently association with the increased incidence of chronic kidney diseases.

### 2.4 In Vivo Transient H_2_O_2_ Fluctuation Imaging by Cy‐Mu‐7 Based Nanoprobe

**Figure 6:**
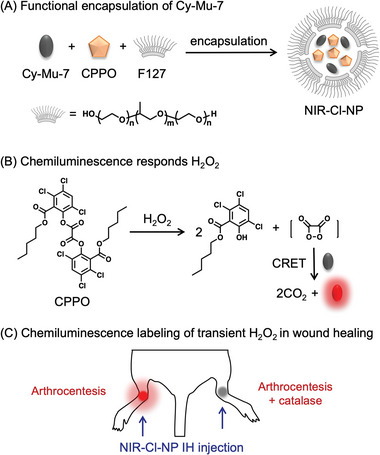
Noise free H_2_O_2_ detection. A) Illustration of NIR‐Cl‐NP. B) The mechanism of the chemiluminescence H_2_O_2_ detection. C) NIR‐Cl‐NP for H_2_O_2_ dynamic imaging in vivo.

H_2_O_2_ has long been recognized as an important endogenous signaling molecule that modulates physiological processes such as stem cell growth, proliferation, and differentiation.^[^ ^41^, ^42^ ^]^ Since the Niethammer group visualized the H_2_O_2_ production at the wound margin of zebrafish and illustrated its signaling roles to recruit immune cells for wound regeneration based on a genetically encoded H_2_O_2_ fluorescent probe in 2009, the research on relevant physiological processes at the small animal level has been stagnant due to the lack of efficient fluorescent probes with high SNR for in vivo imaging.^[^ ^43^, ^44^, ^45^ ^]^ Chemiluminescence‐based detection system is a preferred option to realize ultra‐high SNR H_2_O_2_ imaging because of the noise‐free feature.^[^ ^46^, ^47^ ^]^ As shown in **Figure** **6**, bis[3,4,6‐trichloro‐2‐(pentyl‐oxycarbonyl)phenyl]oxalate (CPPO) reacted with H_2_O_2_ to produce a specific active intermediate named 1,2‐dioxetanedione which was further degraded spontaneously and transferred chemical energy to the nearby fluorophore through CRET.^[^ ^18^, ^48^ ^]^ The Zhang group reported that the energy gap between the LUMO of the 1,2‐dioxetanedione and the HOMO of the dyes should be ranged between 1.20 and 5.01 eV to realize efficient CRET.^[^ ^18^ ^]^ Supported by our aforementioned spectral and computational results of Cy‐Mu‐7, we speculated that Cy‐Mu‐7 as a proper acceptor might facilitate the NIR chemiluminescence H_2_O_2_ detection to avoid the generally used inefficient multi‐stage coupling of CRET and fluorescence resonance energy transfer.

**Figure 7:**
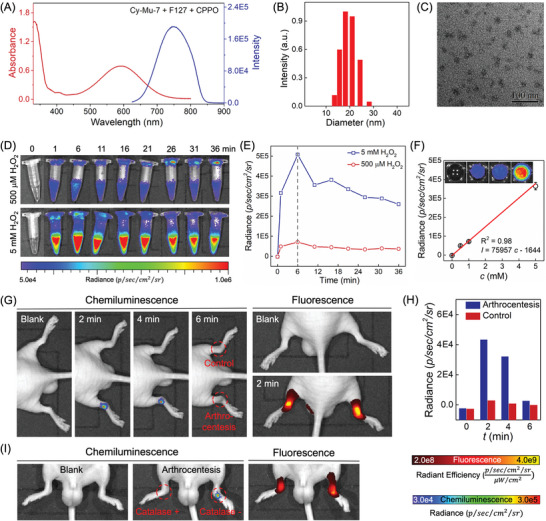
In vivo transient H_2_O_2_ fluctuation imaging. A) UV–vis absorption and fluorescence spectra of 5 mg mL^−1^ NIR‐Cl‐NP in water. B) DLS and C) TEM image of NIR‐Cl‐NP. D) Time dependent chemiluminescence responses of 5 mg mL^−1^ NIR‐Cl‐NP toward 500 µm H_2_O_2_ and 5 mm H_2_O_2_ in water at 37 °C. E) Corresponding chemiluminescence intensity changes of (D). F) Quantitative analysis of H_2_O_2_ by 5 mg mL^−1^ NIR‐Cl‐NP. The chemiluminescence intensities were obtained 6 min after H_2_O_2_ addition at 37 °C. Inset: Corresponding chemiluminescence images obtained by an IVIS with Cy5.5 filter. G) Synchronous fluorescence imaging and chemiluminescence imaging of H_2_O_2_ production and fluctuation in mice after arthrocentesis. 5 µL 10 mg mL^−1^ NIR‐Cl‐NP was injected via hypodermic injection. H) Corresponding chemiluminescence radiance changes of (G). I) Synchronous fluorescence imaging and chemiluminescence imaging of H_2_O_2_ in mice after arthrocentesis with or without the presence of 2000 unit/mL catalase. *λ*_ex_ = 610 nm; slit 6/6 nm. Error bars represent standard deviations obtained from three independent experiments.

Thus, we encapsulated the Cy‐Mu‐7 and CPPO by Pluronic F‐127 to construct a nanoprobe named NIR‐chemiluminescence‐nanoprobe (NIR‐Cl‐NP).^[^ ^18^, ^49^, ^50^ ^]^ NIR‐Cl‐NP displayed similar optical properties in aqueous solution with the Cy‐Mu‐7‐HSA system and the average diameters were ≈20 nm as determined by dynamic light scattering and transmission electron microscopy (**Figure** **7**A–C). To evaluate the chemiluminescence H_2_O_2_ detection properties of NIR‐Cl‐NP, we then performed time‐dependent chemiluminescence imaging experiments upon addition of H_2_O_2_ to a NIR‐Cl‐NP containing centrifuge tube at 37 °C (Figure 7D,E). The chemiluminescence intensity of the reaction system enhanced sharply within the first 1 min and peaked after 6 min of reaction with a 70 000‐fold enhancement even under the appearance of 500 µm H_2_O_2_. Besides, the chemiluminescence intensities were linearly correlated with the concentrations of H_2_O_2_ (Figure 7F). These results demonstrated that NIR‐Cl‐NP could detect H_2_O_2_ quantificationally with ultra‐high SNR chemiluminescence signal.

Supported by the in vitro spectroscopic and imaging analysis results, we further evaluated the possibility of NIR‐Cl‐NP to image the dynamic of H_2_O_2_ in mice during injury. After subcutaneous injection of NIR‐Cl‐NP into the joints of mice hindlimbs, we used a disposable syringe to pierce one of the joints to induce tissue trauma. After that, time‐dependent chemiluminescence imaging and fluorescence imaging were immediately performed by an IVIS. Fluorescence images illustrated the distribution of NIR‐Cl‐NP in mice. The chemiluminescence intensities enhanced significantly in the pierced side compared with the control side (Figure 7G). At the same time, the H_2_O_2_ surged at the pierced site within 2 min and then decreased rapidly (Figure 7H). Compared with the reported dynamic of H_2_O_2_ at the wound margin in zebrafish, the chemiluminescence intensity decrease might be caused by the fast diffusion of H_2_O_2_ in tissues of mice.^[^ ^43^, ^51^ ^]^ To further verify that the chemiluminescence signals were caused by the endogenic H_2_O_2_, we added 2000 unit mL^−1^ catalase to the solution of NIR‐Cl‐NP and injected subcutaneously to the joints of mice hindlimbs. As shown in Figure 7I, the presence of catalase inhibited the chemiluminescence signal significantly after arthrocentesis. Thus, as far as we know, NIR‐Cl‐NP as a robust probing system realized the in vivo visualization of transient H_2_O_2_ fluctuation at the mouse level for the first time.

## 3 Conclusion

In conclusion, we successfully transformed traditional cyanine‐7 dyes via asymmetrical introduction of a pyridine group to obtain new cyanine dyes with activable NIR fluorescence signals and enlarged Stokes shift (≈230 nm). These improvements led to an increased SNR when applied to in vivo imaging applications. For instance, using the present Cy‐Mu‐7 dyes, we were able to achieve ultra‐fast and high contrast in vivo kidney labeling in mice, as well as the fluorescence‐based detection of acute kidney injury induced by nephrotoxic drugs. We found that liver failure exacerbated renal clearance of the dye, leading us to suggest that a change in clearance pathways might underpin the association between non‐alcoholic fatty liver disease and increased incidence of chronic kidney disease. To functionalize the dyes and construct noise‐free imaging tools, we encapsulated the chemiluminescence reaction moiety of H_2_O_2_ and Cy‐Mu‐7 by Pluronic F‐127 to obtain nanoprobe for H_2_O_2_ detection that operates with ultra‐high SNR. This nanoprobe allowed transient production and fluctuation of endogenous H_2_O_2_ in a murine model for emergency trauma to be observed readily. The present study provides a roadmap for improving the performance improvement of fluorescent probes. This, in turn, might allow the development of an increased understanding of cell processes in situ using relatively non‐perturbing, optical‐based analysis methods.

## 4 Experimental Section

### Materials

Albumin from rat serum was purchased from Sigma‐Aldrich. Bovine serum albumin was purchased from Innochem. Albumin human was purchased from Psaitong. The 4–12% SDS‐PAGE Precast Gel, Coomassie Blue Super‐Fast Staining Solution and the related materials were all purchased from Beyotime. All chemicals were purchased from commercial suppliers and used without further purification. All solvents were purified prior to use. Distilled water was used after passing through a water ultra‐purification system. TLC analysis was performed using precoated silica plates. Edinburgh FS5 and Hitachi F−7000 fluorescence spectrophotometer were employed to measure fluorescence spectra. Edinburgh SC‐30 integrating sphere was used to measure the absolute quantum yields of the dyes. Hitachi U‐3900 UV–vis spectrophotometer was employed to measure UV–vis spectra. Shanhai Huamei Experiment Instrument Plants provided a PO‐120 quartz cuvette (10 mm). ^1^H NMR and ^13^C NMR experiments were performed with a BRUKER AVANCE III HD 600 and 151 MHz NMR spectrometer, respectively (Bruker, Billerica, MA). Coupling constants (*J* values) are reported in hertz. HR‐MS determinations were carried out on a Thermo Scientific Q Exactive Instrument. The BIO‐RAD Mini‐PROTEAN Tetra Cell was used in the electrophoresis experiments. The corresponding fluorescent images and Coomassie blue staining images were obtained by a BIO‐RAD ChemiDoc XRS+ Imaging System. The in vivo imaging experiments and ex vivo imaging experiments were performed with an IVIS Lumina LT Series III system. Cell imaging experiments were performed with a ZEISS LSM‐710 Confocal Fluorescence Microscopy. The TEM image was obtained by a JEM‐2100 Transmission Electron Microscope. Zetasizer Nano ZS nanoparticle analyzer was used in the DLS analysis experiments. Male 6‐week‐old BALB/c‐nu mice were purchased from SPF (Beijing) Biotechnology Co., Ltd. Production Permit No.: SCXK (Beijing) 2019‐0010. Male 6‐week‐old ICR mice were purchased from Shanxi Jetman Biotechnology Co., Ltd. Production Permit No.: SCXK (Shanxi) 2019‐0010. This study was performed in strict accordance with the Chinese guidelines for the care and use of laboratory animals and was approved by the Institutional Animal Care and Use Committee of Scientific Research in Shanxi University (SXULL2019033).

### Synthesis of the Dyes

All of the synthesis procedures and characterization data were presented in the supporting information.

### Preparation of NIR‐Cl‐NP

The preparation of the NIR‐Cl‐NP was based on the reported procedure by the Zhang group.^[^ ^18^ ^]^

### Preparation of Solutions of Dyes and Analytes

Stock solution of the dyes (2 mm) was prepared in DMSO. Stock solutions of other analytes were prepared by direct dissolution in deionized water. All chemicals used were of analytical grade.

### General Fluorescence Spectra Measurements

The detection experiments were measured in PBS (pH 7.4, 10 mm). Typically, into a 950 µL PBS solution, 50 µL albumin solution (20 mg mL^−1^) and 5 µL dye were added. The fluorescence spectra were obtained by fluorescence spectrometer 10 min after mixing.

### Theoretical Calculation

The DFT and TD‐DFT were employed to understand the structural and electronic properties of Cy‐7 and Cy‐Mu‐7 in the Gaussian 16 A code.^[^ ^19^ ^]^ The molecular structures of Cy‐7 and Cy‐Mu‐7 in the ground state were optimized at M06‐2X/6‐31G(d) level.^[^ ^52^ ^]^ Besides, the solvent effect (in water) was included in all calculations using the polarizable continuum model (PCM). The stable structures have been verified via frequency analysis without imaginary frequency. A localized orbital locator (LOL‐*π*) has been applied to investigate the molecular characteristic of *π* electrons using Multiwfn 3.7.^[^ ^22^, ^23^ ^]^ The maximum absorption wavelength of Cy‐7 and Cy‐Mu‐7 were calculated using an empirical equation given by Nakano.^[^ ^53^ ^]^

### Molecular Docking

The ligand‐removed crystal structure of HSA (PDB ID: 6R7S) was taken from the Brookhaven Protein Data Bank (http://www.rcsb.org/pdb). The AutoGrid was used to calculate Grids. The grid spacing was 0.375 Å as default. Flexible ligand docking was performed by AutoDock 4.2 molecular docking program using the implemented empirical free energy function and the Lamarckian Genetic Algorithm. 2000 docking runs with 2 500 000 energy evaluations were performed. The lowest energy structures of each independent run were clustered with an RMS‐tolerance of 2.0 Å. The output from AutoDock was rendered with PyMol (http://www.pymol.org/).

### Cell Culture and Imaging

The HeLa cells were grown in 1640 medium supplemented with 12% FBS and 1% antibiotics at 37 °C in humidified environment of 5% CO_2_. Cells were plated on a 6‐well plate with slides and allowed to adhere for 24 h. Before the experiments, cells were washed with PBS 3 times. The fluorescence images were obtained by a ZEISS LSM‐710 Confocal Fluorescence Microscopy.

### Ex vivo Chemiluminescence Imaging of Hydrogen Peroxide

5 mg mL^−1^ NIR‐Cl‐NP was added in the centrifuge tube and stock solution of hydrogen peroxide was then added with a final concentration of 500 µm or 5 mm. The corresponding chemiluminescence intensities were monitored by an IVIS. Emission filter: Cy5.5.

### In Vivo Fluorescence Imaging

For a general real‐time fluorescence imaging, 6‐week‐old BALB/c‐nu mice without treatment were imaged by an IVIS Lumina LT Series III system as the blank control. After IV injection of a PBS solution containing 160 µm Cy‐Mu‐7 and 10 mg mL^−1^ HSA, the mice were imaged under the system upon isoflurane anesthesia immediately. For the Adriamycin induced AKI model imaging, 10 mg kg^−1^ Adriamycin (a water solution, 100 µL/20 g body weight) was injected intravenously into mice. 5 d after Adriamycin injection, the mice were injected with a PBS solution containing 160 µm Cy‐Mu‐7 dyes and 10 mg mL^−1^ HSA intravenously and imaged under the system upon isoflurane anesthesia immediately. Excitation filter: 605 nm; Emission filter: Cy5.5.

### Protein Concentration Analysis in Urine

Healthy mice and 10 mg kg^−1^ Adriamycin injected 6‐week‐old BALB/c‐nu mice were domesticated and spontaneously voided urine samples were collected every day. 10 µL of the urine samples were added to the BCA Protein Assay Kit and analyzed according to the standard method.

### Ex Vivo Mouse Imaging

6‐week‐old male ICR mice were injected intraperitoneally with saline as the control group, 24 mg APAP containing 0.5% CMC suspension as the APAP group, 16 mg NAC containing saline and further 24 mg APAP containing 0.5% CMC suspension 1 h after NAC injected as the NAC‐APAP group, 24 mg APAP containing 0.5% CMC suspension and further 16 mg NAC containing saline 1 h after APAP injected as the APAP‐NAC group, respectively. PBS solution containing 160 µm Cy‐Mu‐7 and 10 mg mL^−1^ HSA were IV injected 24 h after APAP injection and the kidney and liver organs were harvest 5 h after dye injection and imaged immediately.

Detailed synthesis of dyes was presented in Supporting Information.

## Conflict of Interest

The authors declare no conflict of interest.

## Supporting information

Supporting Information

## Data Availability

Research data are not shared.
