# Supplementary material for: Enlarging the Stokes Shift by Weakening the π‐Conjugation of Cyanines for High Signal‐to‐Noise Ratiometric Imaging
Source: Adv Sci (Weinh). 2022 Nov 24;10(3):2205080. doi: 10.1002/advs.202205080 (PMC9875656; doi:10.1002/advs.202205080)
Supplement: Supplementary file 1 — Supporting Information [file ADVS-10-2205080-s001.pdf]

Supplementary Information

Enlarging the Stokes Shift by Shortening the π-Conjugation of Cyanines for High Signal-to-Noise Ratiometric Imaging

*Yongkang Yue, Tingting Zhao, Zhou Xu, Weijie Chi, Xiaojun Chai, Jiahong Ai, Jiawei Zhang, Fangjun Huo, Robert M. Strongin, Caixia Yin**

This PDF file includes:

Supplementary text
Figures S1 to S20
SI Reference

Supplementary text

Synthesis of dyes

Scheme S1. Synthesis of **Indole-Cy-7**, **Pyridine-Cy-7** and **Semi-Cy-7**.

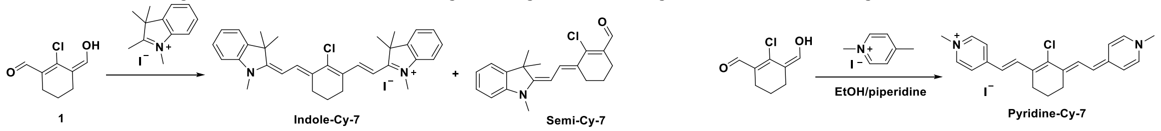

Indole-Cy-7 was synthesized based on the reported procedure.[1] ^{1}H NMR (600 MHz, DMSO-d_6) δ 8.25 (d, *J* = 14.2 Hz, 2H), 7.63 (d, *J* = 7.4 Hz, 2H), 7.45 (d, *J* = 6.0 Hz, 4H), 7.34 – 7.26 (m, 2H), 6.31 (d, *J* = 14.2 Hz, 2H), 3.70 (s, 6H), 2.72 (t, *J* = 5.7 Hz, 4H), 1.91 – 1.83 (m, 2H), 1.68 (s, 12H). ^{13}C NMR (151 MHz, DMSO) δ 173.12, 148.14, 143.33, 143.15, 141.47, 129.02, 126.54, 125.61, 122.86, 111.90, 102.36, 49.35, 32.04, 27.80, 26.36.

Semi-Cy-7. 0.301 g (1 mmol) 1,2,3,3-tetramethyl-3H-indol-1-ium and 1 equiv. **compound 1** was added to 20 mL EtOH, the mixture was reflux for 6 h to complete the reaction. EtOH was separated under reduced pressure and the residue was separated by column chromatography using MeOH/DCM (1/10) as eluent to obtain **Semi-Cy-7** as a red powder. ^{1}H NMR (600 MHz, DMSO-d_6) δ 10.12 (s, 1H), 7.78 (d, *J* = 13.1 Hz, 1H), 7.35 (d, *J* = 7.3 Hz, 1H), 7.22 (td, *J* = 7.7, 1.2 Hz, 1H), 6.97 (d, *J* = 7.9 Hz, 1H), 6.94 (t, *J* = 7.4 Hz, 1H), 5.60 (d, *J* = 12.9 Hz, 1H), 3.28 (s, 3H), 2.60 (t, *J* = 5.7 Hz, 2H), 2.38 (t, *J* = 6.0 Hz, 2H), 1.68 (dd, *J* = 10.2, 4.0 Hz, 2H), 1.59 (s, 6H).

Pyridine-Cy-7. Similar to the synthesis of **Indole-Cy-7**, piperidine was added as the catalyst in the system. ^{1}H NMR (600 MHz, DMSO-d_6) δ 7.80 (d, *J* = 6.8 Hz, 4H), 7.73 (d, *J* = 14.1 Hz, 2H), 7.17 (d, *J* = 6.8 Hz, 4H), 5.98 (d, *J* = 14.2 Hz, 2H), 3.78 (s, 6H), 2.49 (d, *J* = 6.7 Hz, 4H), 1.74 (p, *J* = 6.3 Hz, 2H). ^{13}C NMR (151 MHz, DMSO) δ 149.14, 141.49, 141.06, 135.64, 121.64, 117.86, 110.09, 44.54, 27.04, 21.50. HR-MS [Pyridine-Cy-7]$^{+}$: m/z Calcd 351.1623, Found 351.1620.

Scheme S2. Synthesis of **Indole-Cy-7**, **Pyridine-Cy-7** and **Semi-Cy-7**.

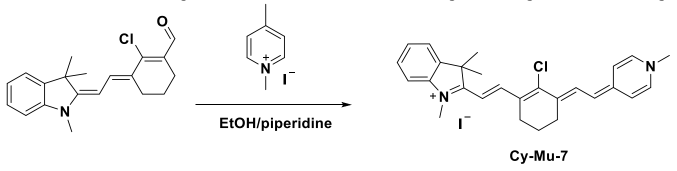

Cy-Mu-7. In a **Semi-Cy-7** (0.065 g, 0.2 mmol) containing EtOH solution, 1 equiv. 1,4-dimethylpyridin-1-ium was added. After the addition of piperidine as the catalyst, the mixture was refluxed for 12 h to complete the reaction. EtOH was separated under reduced pressure and the residue was separated by column chromatography using MeOH/DCM (1/5) as eluent to obtain **Cy-Mu-7** as a dark powder. ^{1}H NMR (600 MHz,

DMSO-d_6) δ 8.68 (d, *J* = 6.4 Hz, 2H), 8.17 – 8.02 (m, 3H), 7.57 (d, *J* = 12.8 Hz, 1H), 7.33 (d, *J* = 7.3 Hz, 1H), 7.20 (t, *J* = 7.7 Hz, 1H), 6.97 – 6.83 (m, 3H), 5.59 (d, *J* = 12.9 Hz, 1H), 4.20 (s, 3H), 3.25 (s, 3H), 2.71 – 2.56 (m, 4H), 1.81 (p, *J* = 6.3, 5.9 Hz, 2H), 1.59 (s, 6H). ^{13}C NMR (151 MHz, DMSO) δ 161.71, 152.83, 144.99, 144.79, 139.67, 139.12, 138.19, 129.75, 128.39, 127.94, 124.37, 123.29, 122.27, 122.22, 120.97, 107.78, 94.06, 47.00, 46.23, 29.72, 28.31, 27.30, 26.20, 21.35.

Scheme S3. Synthesis of **Semi-Cy-7-COOH**, **Cy-Mu-7-COOH** and **Cy-Mu-7-amide**.

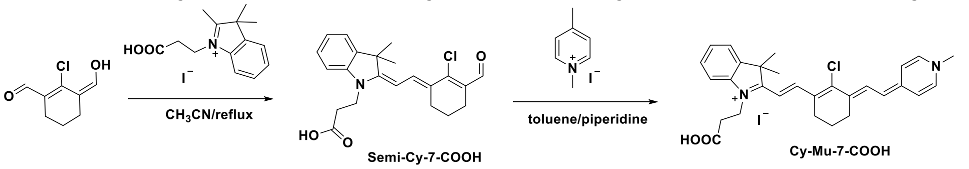

Semi-Cy-7-COOH. 0.360 g (1 mmol) 1,2,3,3-tetramethyl-3H-indol-1-ium and 1 equiv. **compound 1** was added to 20 mL acetonitrile, the mixture was reflux for 6 h to complete the reaction. Acetonitrile was separated under reduced pressure and the residue was separated by column chromatography using MeOH/DCM (1/5, 0.5% glacial acetic acid) as eluent to obtain **Semi-Cy-7-COOH** as a red powder. ^{1}H NMR (600 MHz, Chloroform-*d*) δ 10.24 (s, 1H), 7.80 (d, *J* = 12.6 Hz, 1H), 7.25 – 7.16 (m, 2H), 6.96 (td, *J* = 7.4, 0.9 Hz, 1H), 6.77 (d, *J* = 7.8 Hz, 1H), 5.57 (d, *J* = 12.7 Hz, 1H), 4.05 (t, *J* = 7.3 Hz, 2H), 2.74 (t, *J* = 7.3 Hz, 2H), 2.59 – 2.55 (m, 2H), 2.48 (t, *J* = 6.1 Hz, 2H), 1.77 (p, *J* = 6.2 Hz, 2H), 1.65 (s, 6H).

Cy-Mu-7-COOH. In a **Semi-Cy-7-COOH** (0.076 g, 0.2 mmol) containing methylbenzene solution, 1 equiv. 1,4-dimethylpyridin-1-ium was added. After the addition of piperidine as the catalyst, the mixture was refluxed for 10 h to complete the reaction. The precipitate was filtrated, dried and purified by column chromatography using MeOH/DCM/H_2O (5/2/1) as eluent to obtain **Cy-Mu-7** as a dark powder. ^{1}H NMR (400 MHz, DMSO-d_6) δ 8.66 (d, *J* = 6.5 Hz, 2H), 8.15 – 8.00 (m, 3H), 7.58 (d, *J* = 11.6 Hz, 1H), 7.30 (d, *J* = 7.1 Hz, 1H), 7.18 (td, *J* = 7.8, 1.6 Hz, 1H), 6.95 – 6.77 (m, 3H), 5.70 (d, *J* = 12.7 Hz, 1H), 4.18 (s, 3H), 3.94 – 3.86 (m, 2H), 2.58 (dd, *J* = 10.1, 5.1 Hz, 4H), 2.13 (t, *J* = 7.5 Hz, 2H), 1.83 – 1.76 (m, 2H), 1.56 (s, 6H). [Cy-Mu-7-COOH]$^+$: m/z Calcd 475.2147, Found 475.2048.

Scheme S4. Synthesis of **Semi-Cy-7-ester** and **Cy-Mu-7-ester**.

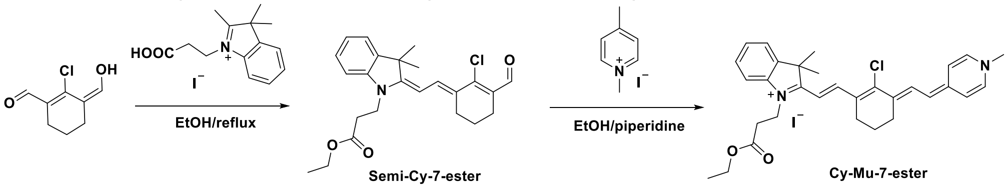

Semi-Cy-7-ester. Similar to the synthesis of **Semi-Cy-7-COOH**, the reaction solvent used EtOH instead, we obtained the **Semi-Cy-7-ester** as a red powder. ^{1}H NMR (600 MHz, Chloroform-*d*) δ 10.26 (s, 1H), 7.80 (d, *J* = 12.6 Hz, 1H), 7.24 – 7.17 (m, 2H), 6.95 (td, *J* = 7.4, 0.9 Hz, 1H), 6.77 (d, *J* = 7.8 Hz, 1H), 5.54 (d, *J* = 12.7 Hz, 1H), 4.12 (q, *J* = 7.1 Hz, 2H), 4.03 (t, *J* = 7.1 Hz, 2H), 2.68 (t, *J* = 7.0 Hz, 2H), 2.60 – 2.57 (m, 2H), 2.48

(t, *J* = 6.1 Hz, 2H), 1.77 (p, *J* = 6.2 Hz, 2H), 1.65 (s, 6H), 1.21 (t, *J* = 7.2 Hz, 3H). HR-MS [Semi-Cy-7-ester + H]$^+$: m/z Calcd 414.1830, Found 414.1830.

Cy-Mu-7-ester. Similar to the synthesis of **Cy-Mu-7**, **Cy-Mu-7-ester** was obtained as a dark powder. ^{1}H NMR (600 MHz, Chloroform-*d*) δ 8.84 (d, *J* = 6.7 Hz, 2H), 8.15 (d, *J* = 15.6 Hz, 1H), 7.80 (d, *J* = 6.8 Hz, 2H), 7.68 (d, *J* = 12.7 Hz, 1H), 7.24 – 7.15 (m, 2H), 6.95 (dd, *J* = 8.1, 6.8 Hz, 1H), 6.76 (d, *J* = 7.8 Hz, 1H), 6.54 (d, *J* = 15.5 Hz, 1H), 5.57 (d, *J* = 12.7 Hz, 1H), 4.48 (s, 3H), 4.12 (q, *J* = 7.2 Hz, 2H), 4.03 (t, *J* = 7.3 Hz, 2H), 2.68 (t, *J* = 7.2 Hz, 2H), 2.58 (q, *J* = 6.8 Hz, 4H), 1.89 (t, *J* = 6.3 Hz, 2H), 1.65 (s, 6H), 1.20 (t, *J* = 7.2 Hz, 3H). ^{13}C NMR (151 MHz, $CDCl_3$) δ 171.28, 160.76, 153.86, 144.01, 143.37, 142.39, 140.19, 139.22, 130.91, 127.94, 127.62, 125.13, 122.84, 121.93, 121.23, 119.87, 107.03, 94.02, 61.09, 47.86, 46.54, 38.41, 31.29, 28.31, 27.16, 26.21, 21.18, 14.09. HR-MS [Cy-Mu-7-ester]$^+$: m/z Calcd 503.2460, Found 503.2468.

Scheme S5. Synthesis of **Semi-Cy-7-benzene** and **Cy-Mu-7-benzene**.

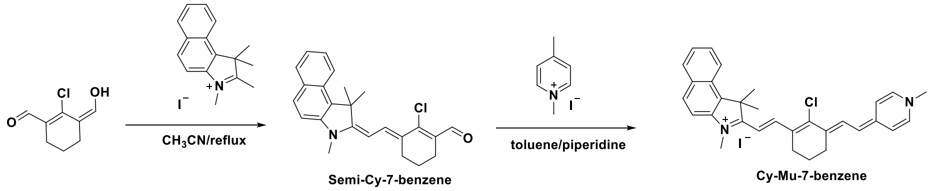

Semi-Cy-7-benzene. ^{1}H NMR (600 MHz, DMSO-d_6) δ 10.12 (s, 1H), 8.11 (d, *J* = 8.6 Hz, 1H), 7.90 (t, *J* = 7.9 Hz, 3H), 7.54 – 7.47 (m, 1H), 7.44 (d, *J* = 8.8 Hz, 1H), 7.31 (t, *J* = 7.5 Hz, 1H), 5.63 (d, *J* = 13.0 Hz, 1H), 3.40 (s, 3H), 2.62 (t, *J* = 6.3 Hz, 2H), 2.39 (t, *J* = 6.1 Hz, 2H), 1.88 (s, 6H), 1.70 (p, *J* = 6.2 Hz, 2H). ^{13}C NMR (151 MHz, DMSO) δ 189.57, 165.41, 147.88, 142.24, 131.60, 131.54, 130.24, 130.22, 130.10, 129.20, 128.66, 127.92, 127.54, 123.13, 122.44, 121.95, 110.63, 93.26, 48.39, 27.55, 26.55, 24.85, 20.97.

Cy-Mu-7-benzene. ^{1}H NMR (600 MHz, DMSO-d_6) δ 8.68 (d, *J* = 6.3 Hz, 2H), 8.09 (q, *J* = 8.4, 6.9 Hz, 4H), 7.88 (t, *J* = 9.3 Hz, 2H), 7.70 (d, *J* = 11.5 Hz, 1H), 7.49 (t, *J* = 7.8 Hz, 1H), 7.41 (d, *J* = 8.7 Hz, 1H), 7.29 (t, *J* = 7.5 Hz, 1H), 6.87 (d, *J* = 15.7 Hz, 1H), 5.62 (d, *J* = 12.9 Hz, 1H), 4.19 (s, 3H), 2.63 (dd, *J* = 13.8, 6.9 Hz, 4H), 1.90 (s, 6H), 1.82 (t, *J* = 6.3 Hz, 2H). ^{13}C NMR (151 MHz, DMSO) δ 163.48, 152.82, 144.97, 142.40, 139.69, 138.18, 130.25, 130.07, 129.58, 129.52, 128.91, 128.73, 127.95, 127.48, 124.34, 123.26, 122.92, 122.20, 121.90, 110.53, 93.87, 48.12, 46.99, 29.97, 27.37, 27.30, 26.22, 21.36. HR-MS [Cy-Mu-7-benzene]$^+$: m/z Calcd 467.2249, Found 467.2247.

Supplementary figures

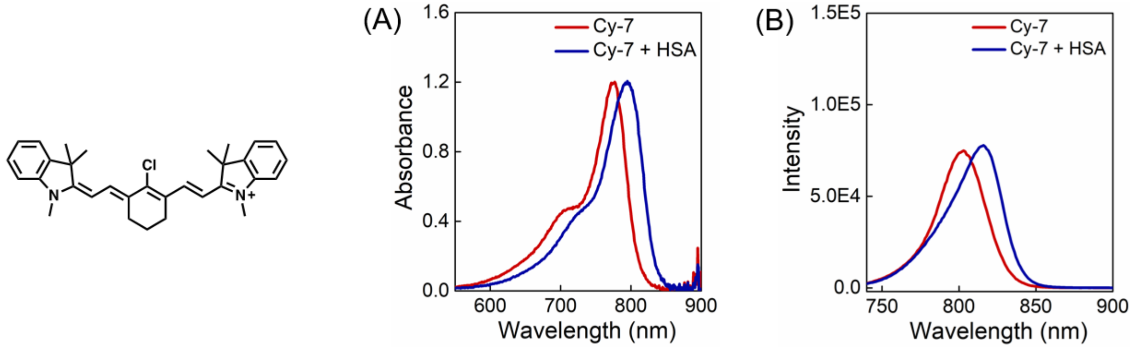

Figure S1. UV-Vis and fluorescence responses of Indole-Cy-7 (10 μM) toward 1 mg/mL HSA in PBS (pH 7.4, 10 mM).

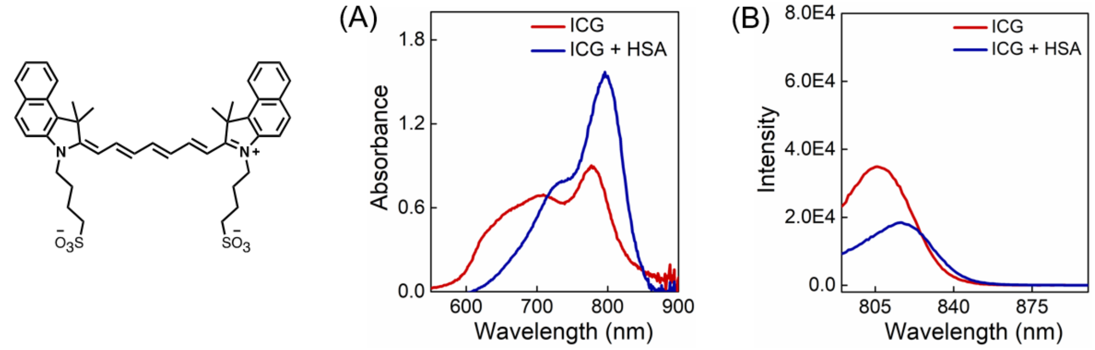

Figure S2. UV-Vis and fluorescence responses of ICG (10 μM) toward 1 mg/mL HSA in PBS (pH 7.4, 10 mM).

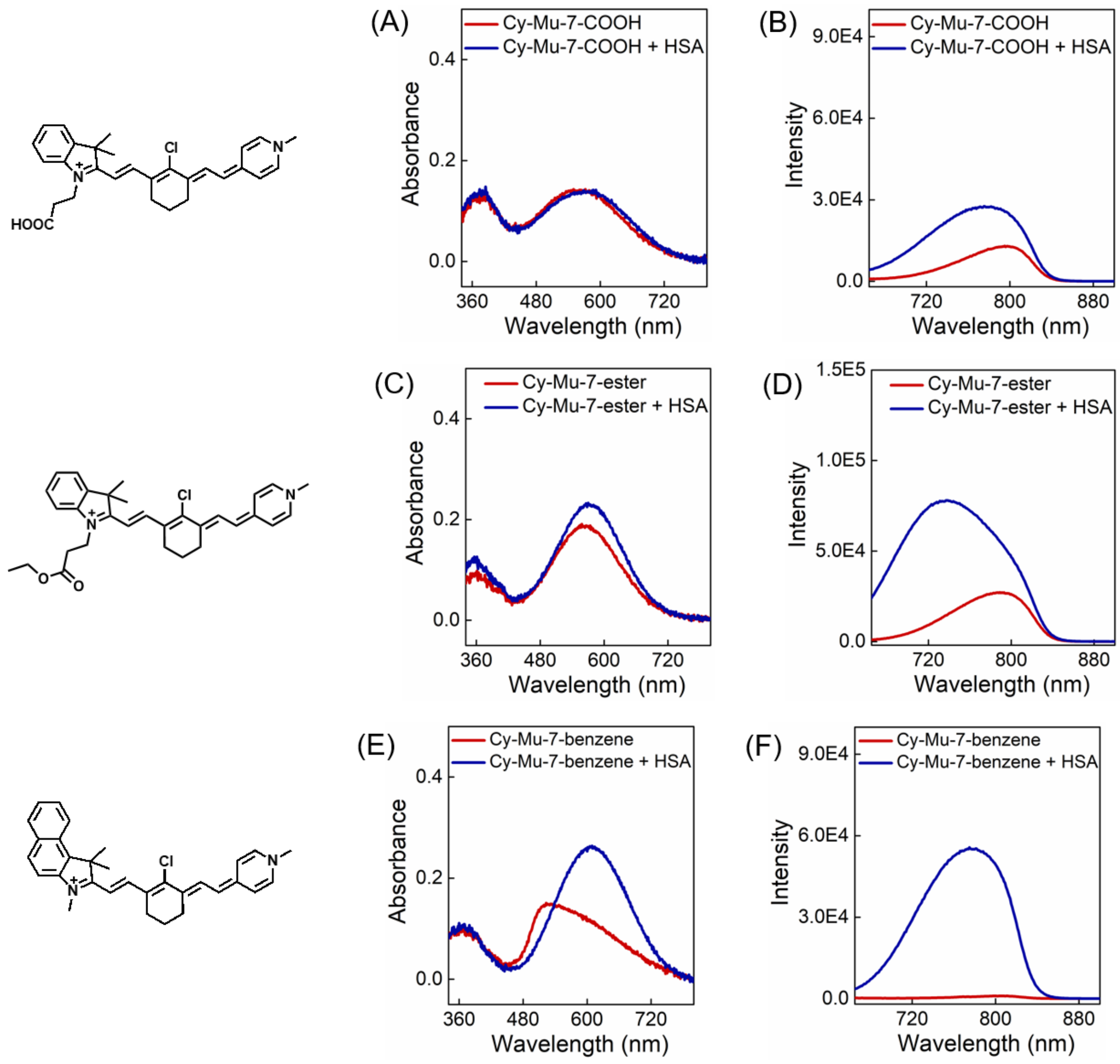

Figure S3. UV-Vis and fluorescence responses of Cy-Mu-7-COOH (λ_{ex} = 600 nm), Cy-Mu-7-ester (λ_{ex} = 580 nm) and Cy-Mu-7-benzene (λ_{ex} = 626 nm) (10 μM) toward 1 mg/mL HSA in PBS (pH 7.4, 10 mM).

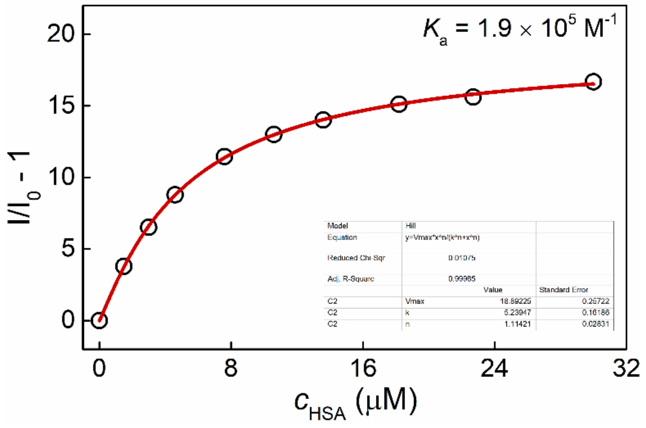

Figure S4. K_a evaluation of Cy-Mu-7 (10 μM) toward HSA in PBS.

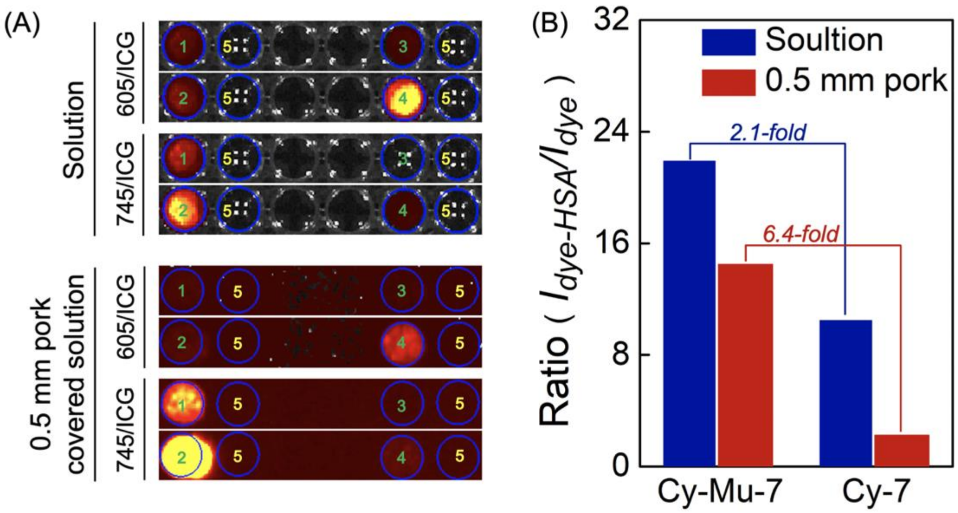

Figure S5. Fluorescence imaging of 100 μL PBS containing 10 μM Cy-Mu-7 or Indole-Cy-7 with or without the presence of HSA. 1. Indole-Cy-7; 2. Indole-Cy-7-HSA; 3. Cy-Mu-7; 4. Cy-Mu-7-HSA; 5. PBS. The 0.5 mm pork slice was obtained by a Microm HM525NX Cryostat Microtome.

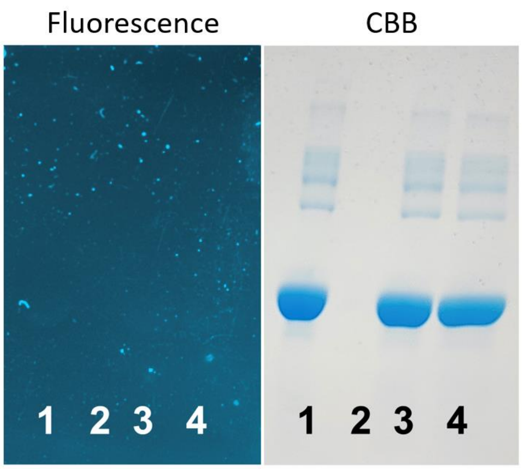

Figure S6. SDS-PAGE Gel analysis of HSA stained with Cy-Mu-7 or Indole-Cy-7. (1) HSA + Cy-Mu-7; (2) blank; (3) HSA + Indole-Cy-7; (4) HSA.

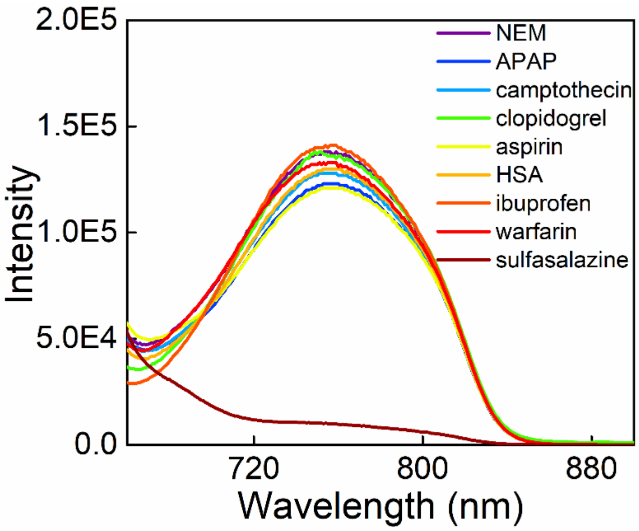

Figure S7. Fluorescence intensity changes of 10 μM Cy-Mu-7 in the presence of 1 mg/mL HSA pre-incubated with 400 μM various compounds including ibuprofen, warfarin, APAP, aspirin, sulfasalazine, camptothecin, clopidogrel and NEM, respectively.

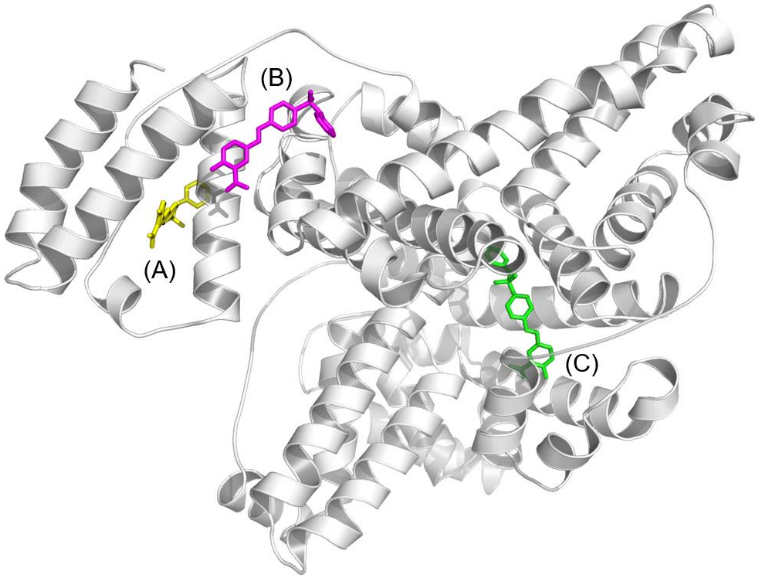

Figure S8. Reported X-ray crystal structure of HSA in complex with sulfasalazine. (PDB ID: 6R7S) Figures of the complexes are generated with PyMol (http://www.pymol.org/).

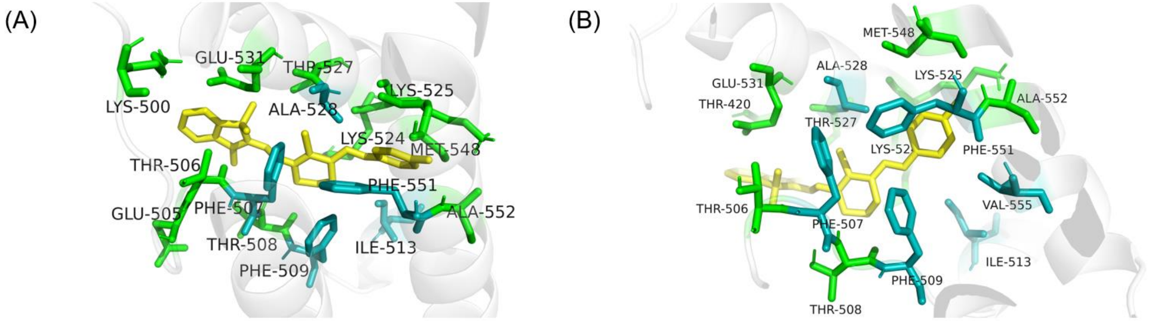

Figure S9. Molecular docking models of Cy-Mu-7 (A) and Cy-Mu-7-ester (B) with domain IIIb of HSA. Figures of the complexes are generated with PyMol (http://www.pymol.org/).

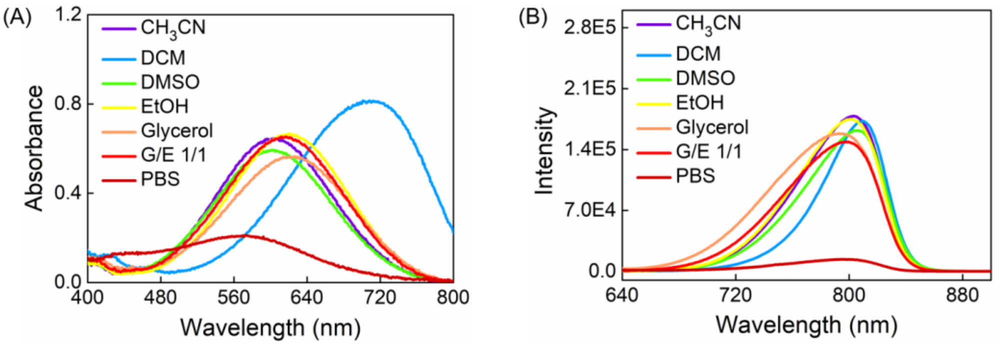

Figure S10. UV-Vis and fluorescence spectra of Cy-Mu-7 (10 μM) in different solvent. G/E 1/1 meant an equal volume mixture of ethanol and glycerol.

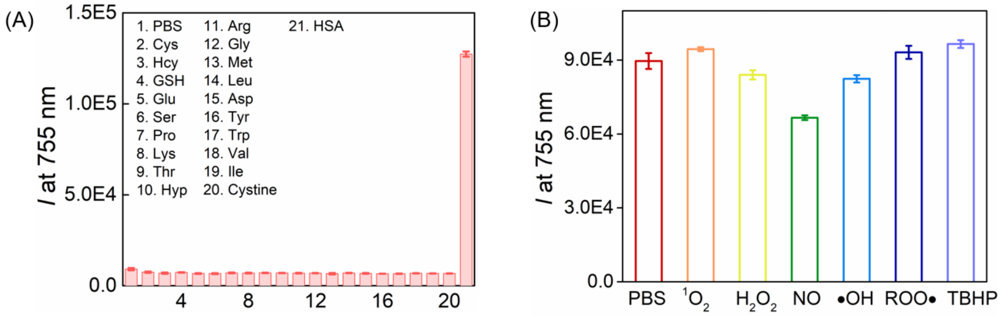

Figure S11. (A) Fluorescence responses of Cy-Mu-7 (10 μM) toward the biological amino acids (HSA 1 mg/mL, Cys 1 mM, GSH 2 mM, Hcy 100 μM, others 200 μM) in aqueous solution. (B) Fluorescence spectra changes of Cy-Mu-7-HSA in the presence of 1 mM various reactive oxygen species.

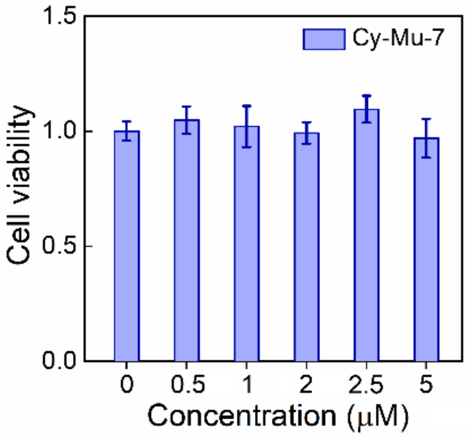

Figure S12. Cell viabilities of HeLa cells incubated with 0-5 μM Cy-Mu-7 for 12 h tested by a CCK-8 assay.

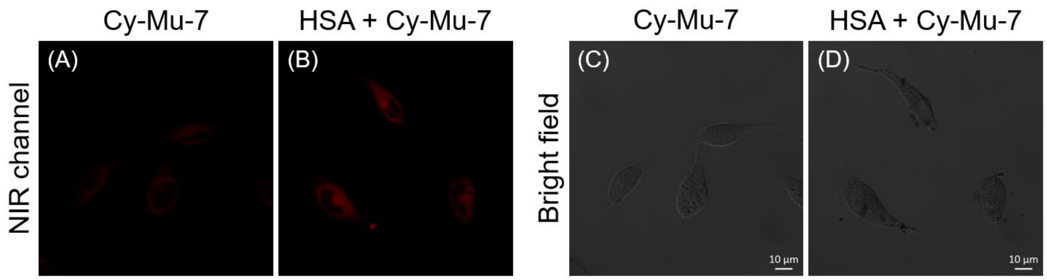

Figure S13. Confocal fluorescence imaging of Cy-Mu-7 labelled HeLa cells. (A) NIR channel fluorescence image of HeLa cells incubated with 5 μM Cy-Mu-7 containing PBS for 15 min and the corresponding bright field image (C). (B) NIR channel fluorescence image of HeLa cells pre-incubated with 1 mg/mL HSA in PBS for 1 h and further incubated with 5 μM Cy-Mu-7 containing PBS for 15 min and the corresponding bright field image (D). NIR channel: λ_{ex} = 633 nm, λ_{em} = 700-750 nm. Scale bar = 10 μm.

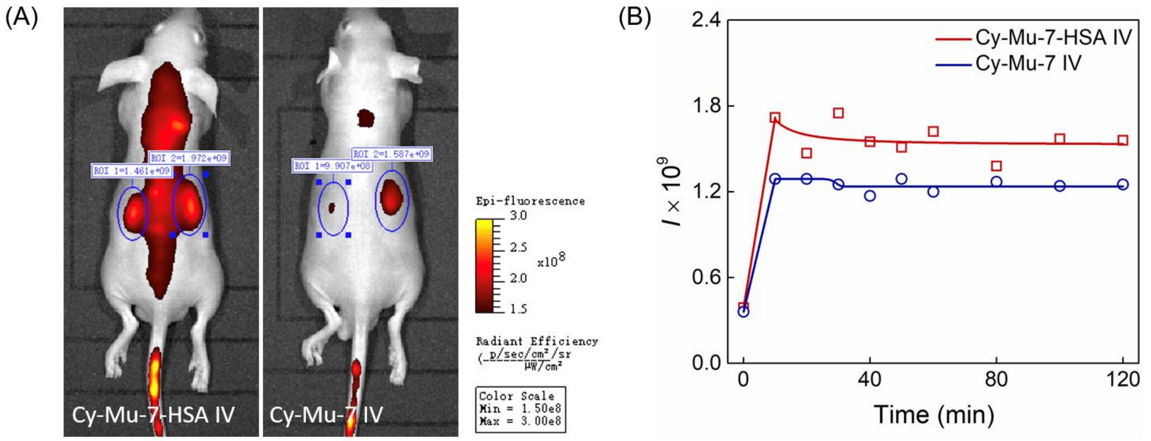

Figure S14. Time dependent fluorescence imaging of mice IV injected with Cy-Mu-7 (160 μM in PBS, 100 μL/20 g) or the pre-bound Cy-Mu-7-HSA (PBS solution containing 160 μM Cy-Mu-7 and 10 mg/mL HSA, 100 μL/20 g body weight). Excitation filter: 605 nm; Emission filter: Cy5.5.

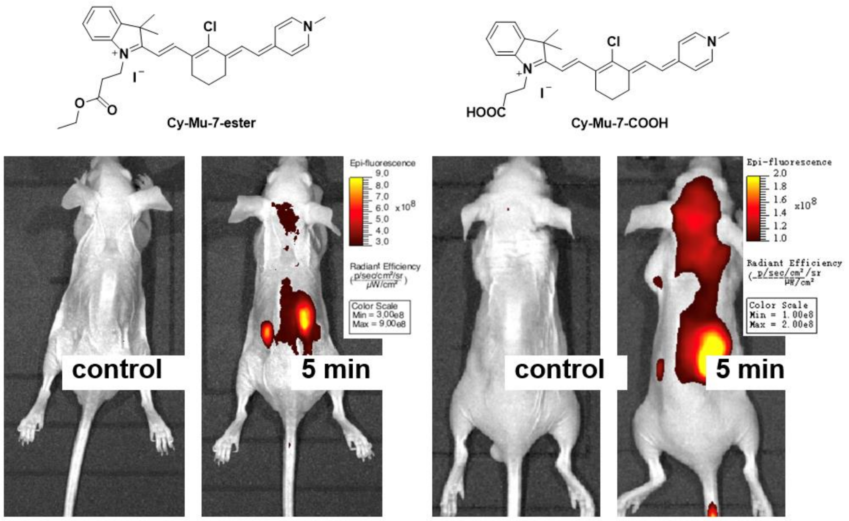

Figure S15. Fluorescence imaging of mice before or 5 min after IV injection of PBS solution containing 160 μM Cy-Mu-7-ester or Cy-Mu-7-COOH and 10 mg/mL HSA. Excitation filter: 605 nm; Emission filter: Cy5.5.

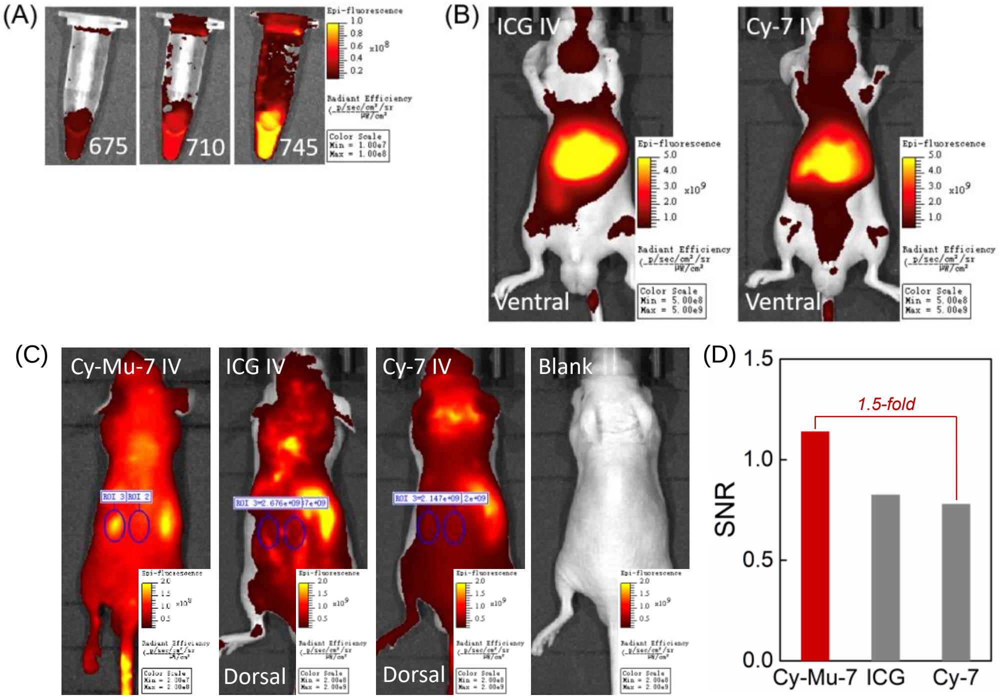

Figure S16. Fluorescence imaging of mice before or 10 min after IV injection of PBS solution containing 160 μM Cy-Mu-7, Indole-Cy-7 or ICG. (A) Fluorescence images of Indole-Cy-7 with different excitation filters. The 745 nm filter brings the best imaging result. (B) Indole-Cy-7 and ICG mainly accumulated in the liver of mice. (C) Comparation of the SNR of the three dyes after IV injection. (D) Corresponding SNR of C. The signal was the fluorescent intensity of the kidney in the blue circle. The noise was

the fluorescent intensity of the ROI in the dorsal. Excitation filter for Cy-Mu-7: 605 nm; Emission filter: Cy5.5. Excitation filter for Indole-Cy-7 and ICG: 745 nm; Emission filter: ICG.

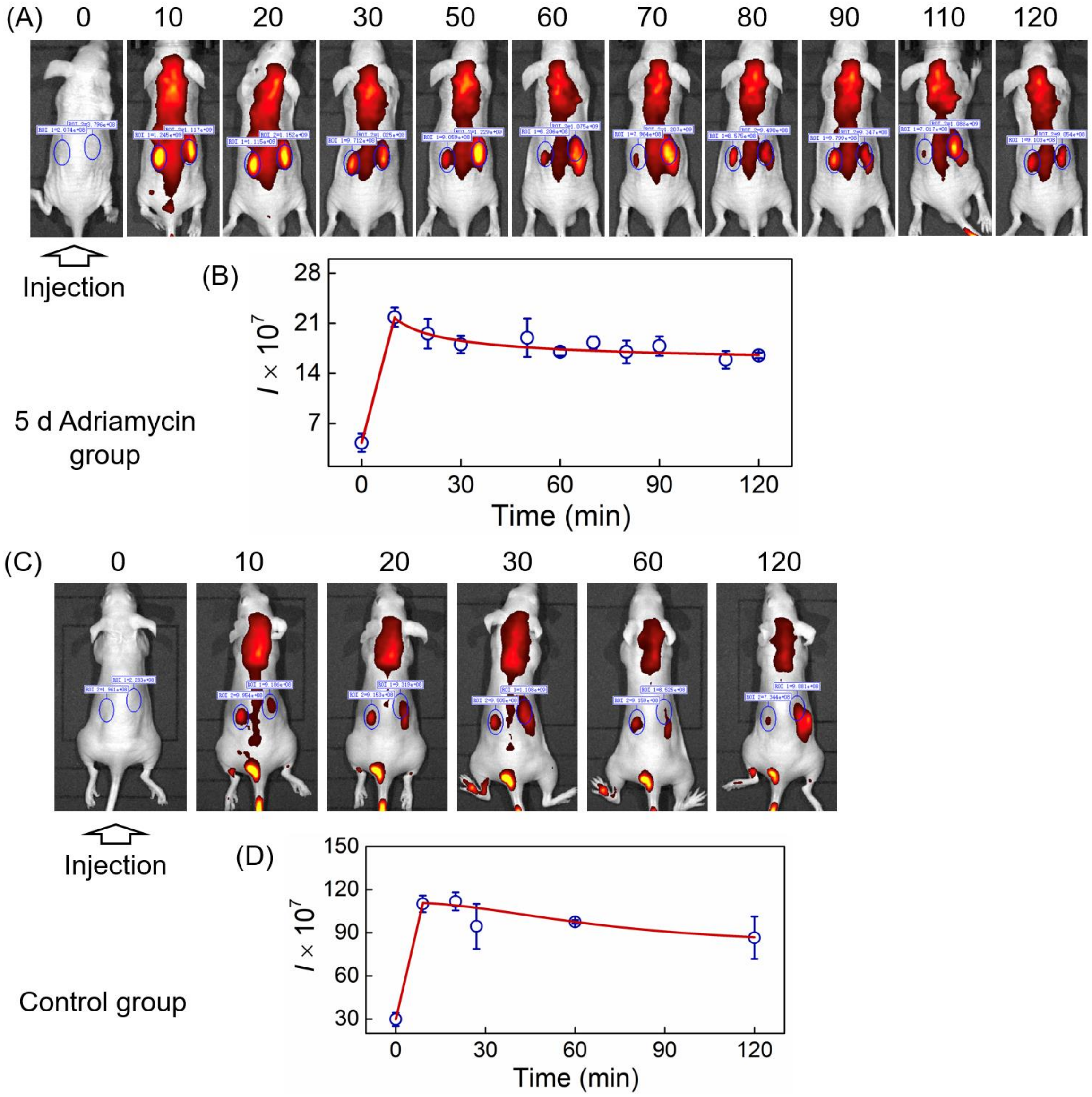

Figure S17. Time dependent fluorescence imaging of AKI (A) and control (C) mice IV injected with PBS solution containing 160 μM Cy-Mu-7-COOH and 10 mg/mL HSA. (B) and (D) were the corresponding fluorescence intensity changes of A and C. Excitation filter: 605 nm; Emission filter: Cy5.5. Error bars represent standard deviations obtained from three independent experiments.

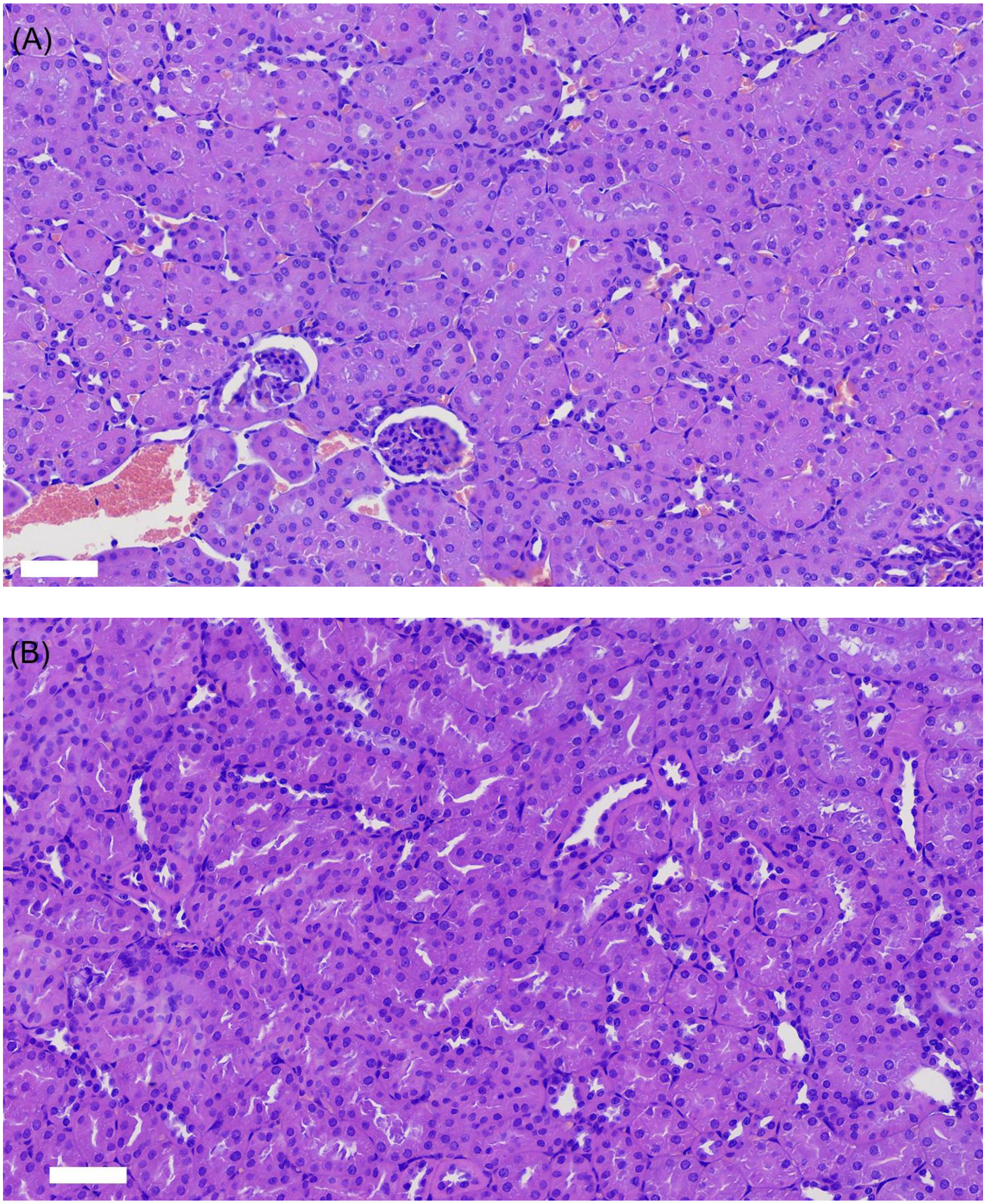

Figure S18. H&E staining of kidney tissues obtained from healthy mice and Adriamycin injected mice after 5 days. (A) Kidney tissue of healthy mice. (B) Kidney tissue of 5-d Adriamycin injected mice.

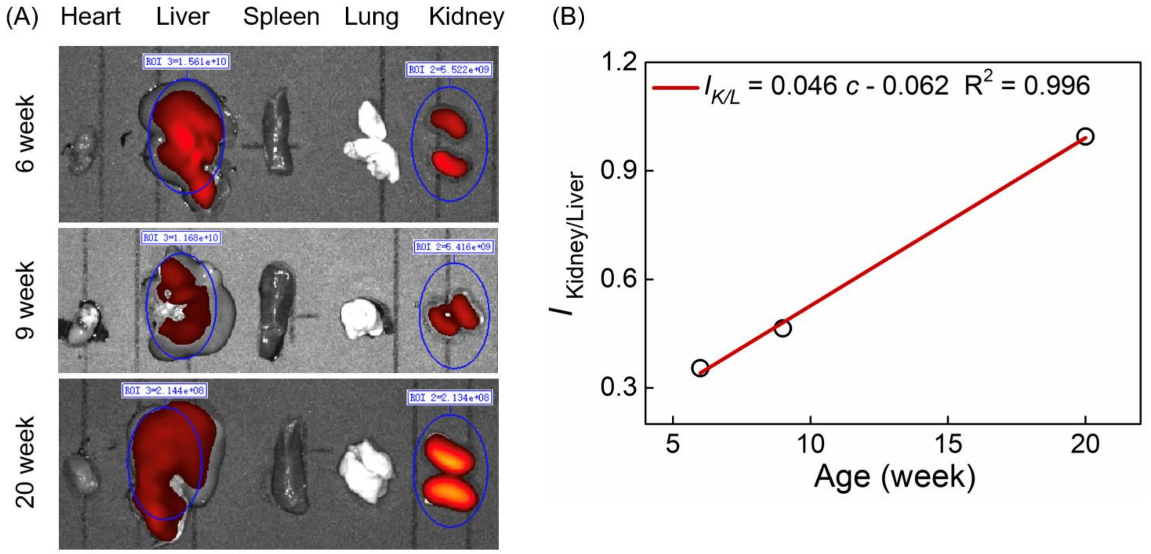

Figure S19. *Ex vivo* fluorescence imaging of organs obtained from 6-week-old, 9-week-old and 20-week-old mice after IV injection of PBS solution containing 160 μM Cy-Mu-7 and 10 mg/mL HSA. Excitation filter: 605 nm; Emission filter: Cy5.5.

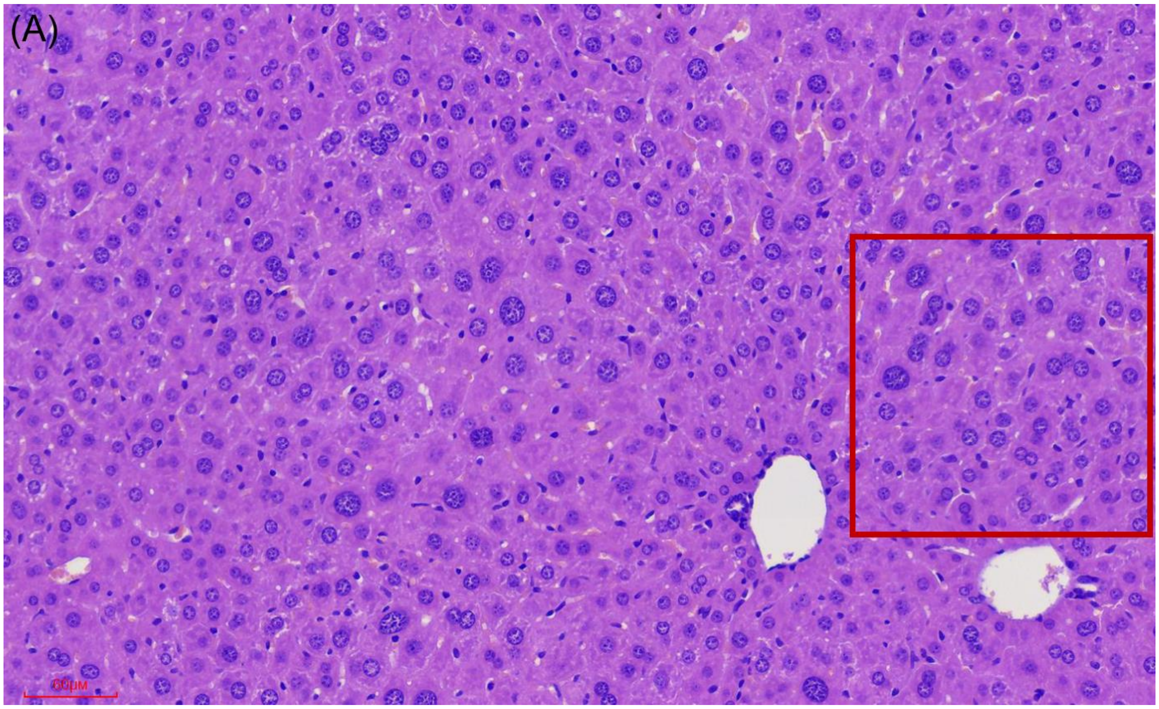

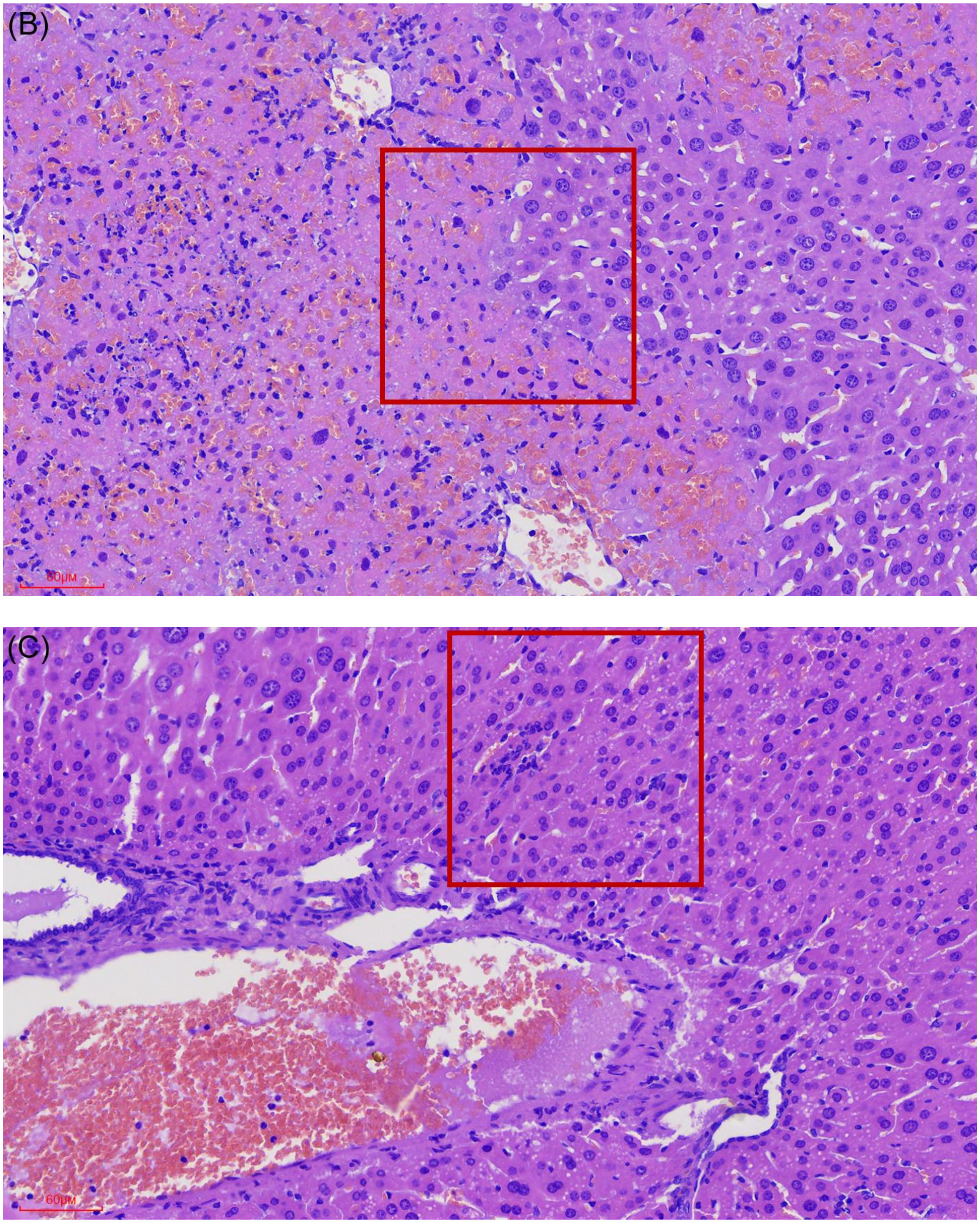

Figure S20. H&E staining of liver tissues obtained from APAP induced acute liver injury mice. Red box labelled the local images presented in the manuscript. (A) Liver tissue of saline IP injected ICR mice. (B) Liver tissue of ICR mice treated with 16 mg NAC and 24 mg APAP 1 h after NAC injection. (C) Liver tissue of ICR mice treated with 24 mg APAP and 16 mg NAC and 1 h after APAP injection.

Characterization

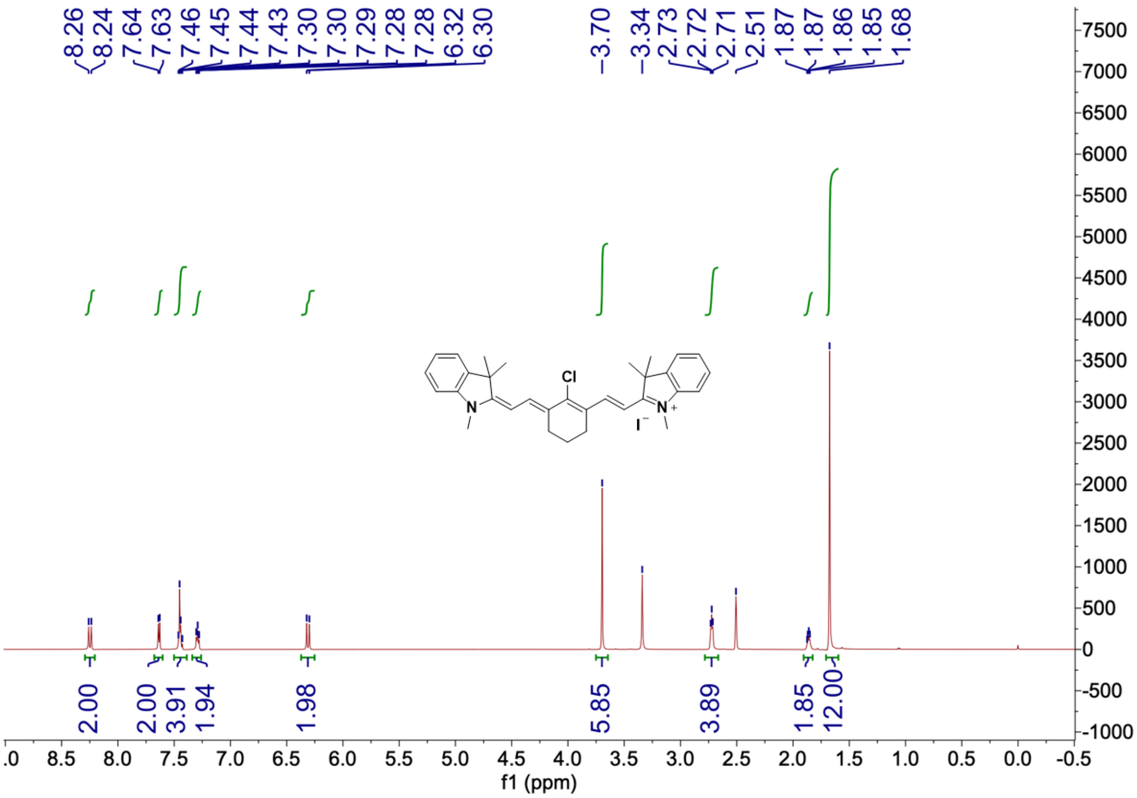

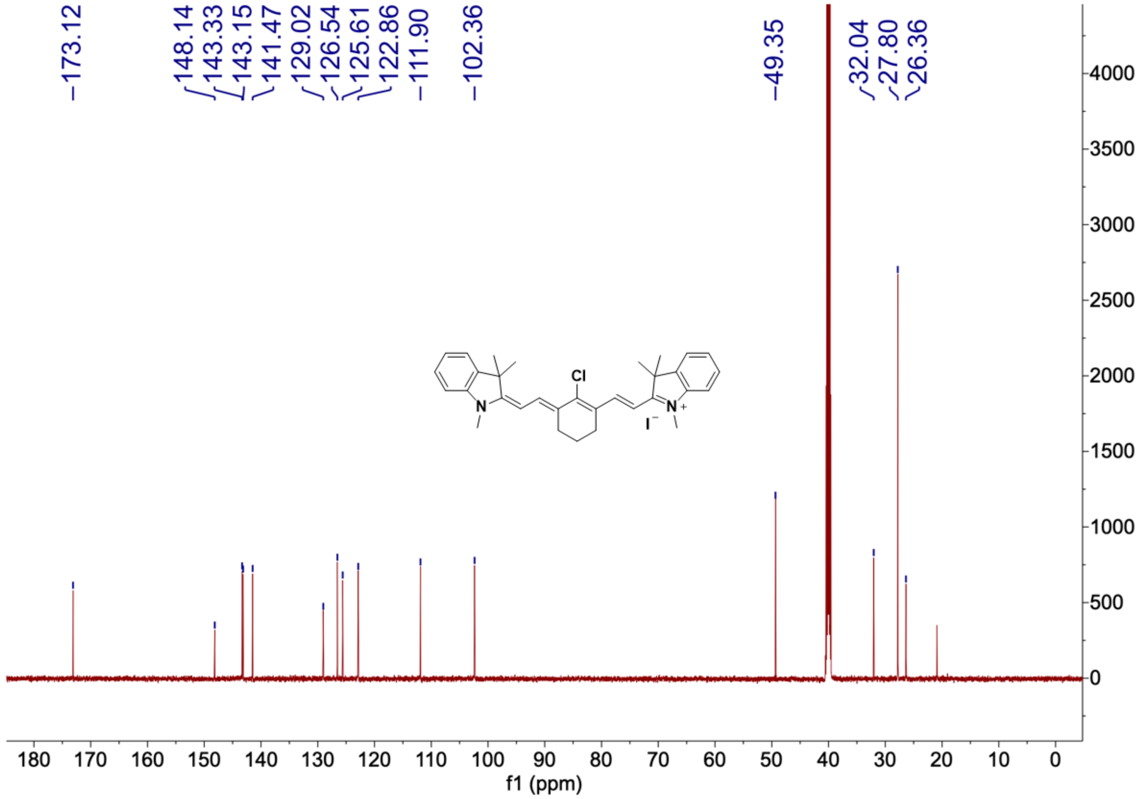

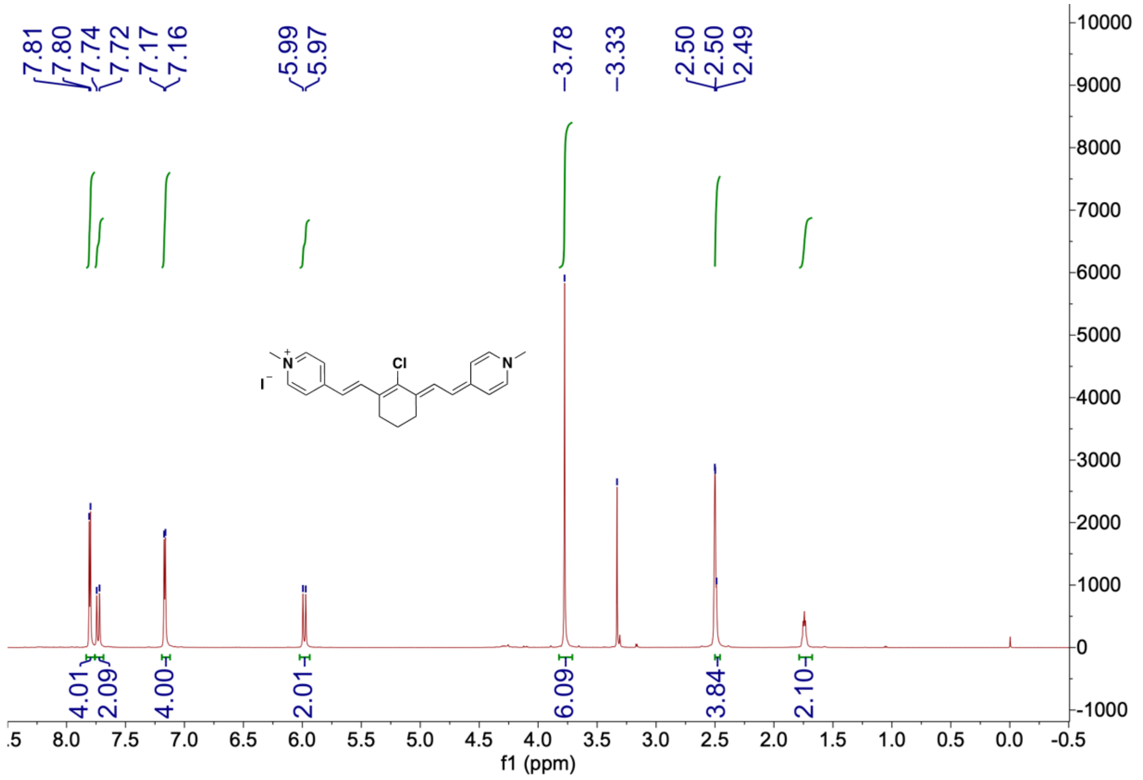
7.81
7.80
7.74
7.72
7.17
7.16
5.99
5.97
3.78
3.33
2.50
2.50
2.49
Cl
N
N
I⁻
4.01
2.09
4.00
2.01
6.09
3.84
2.10
f1 (ppm)

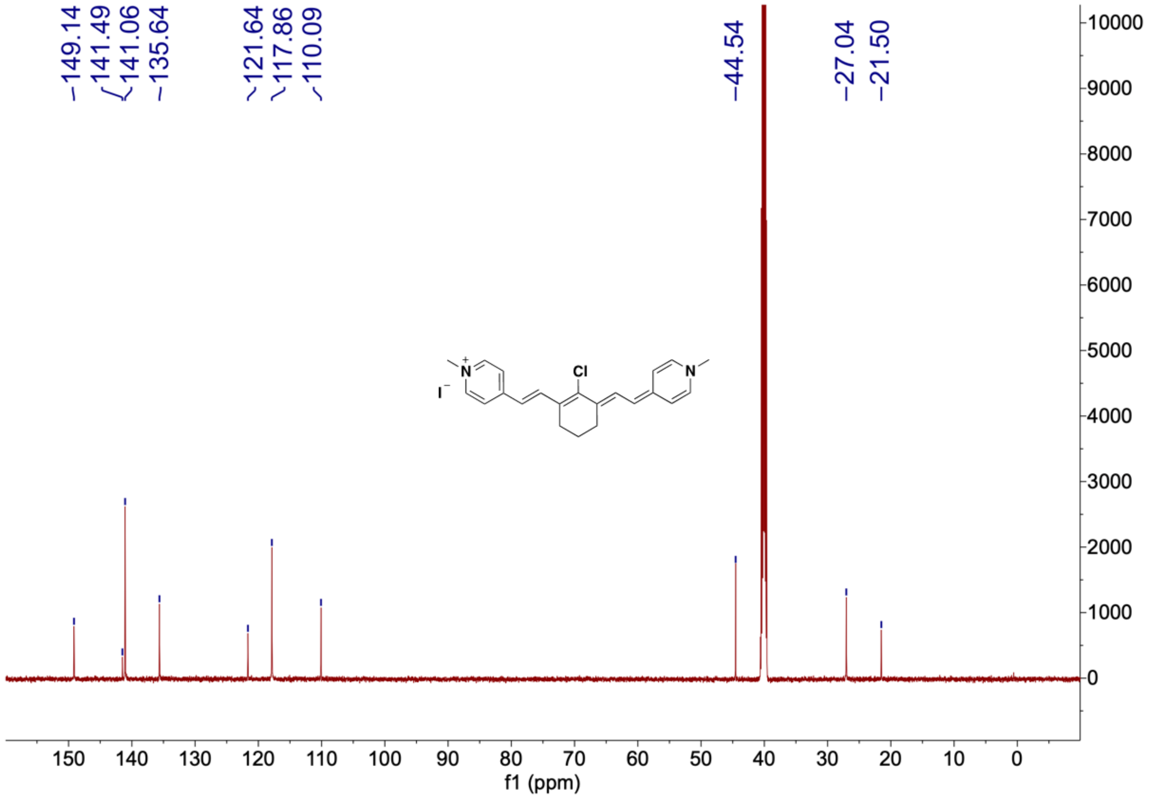
149.14
141.49
141.06
135.64
121.64
117.86
110.09
44.54
27.04
21.50
Cl
N
N
I⁻
f1 (ppm)

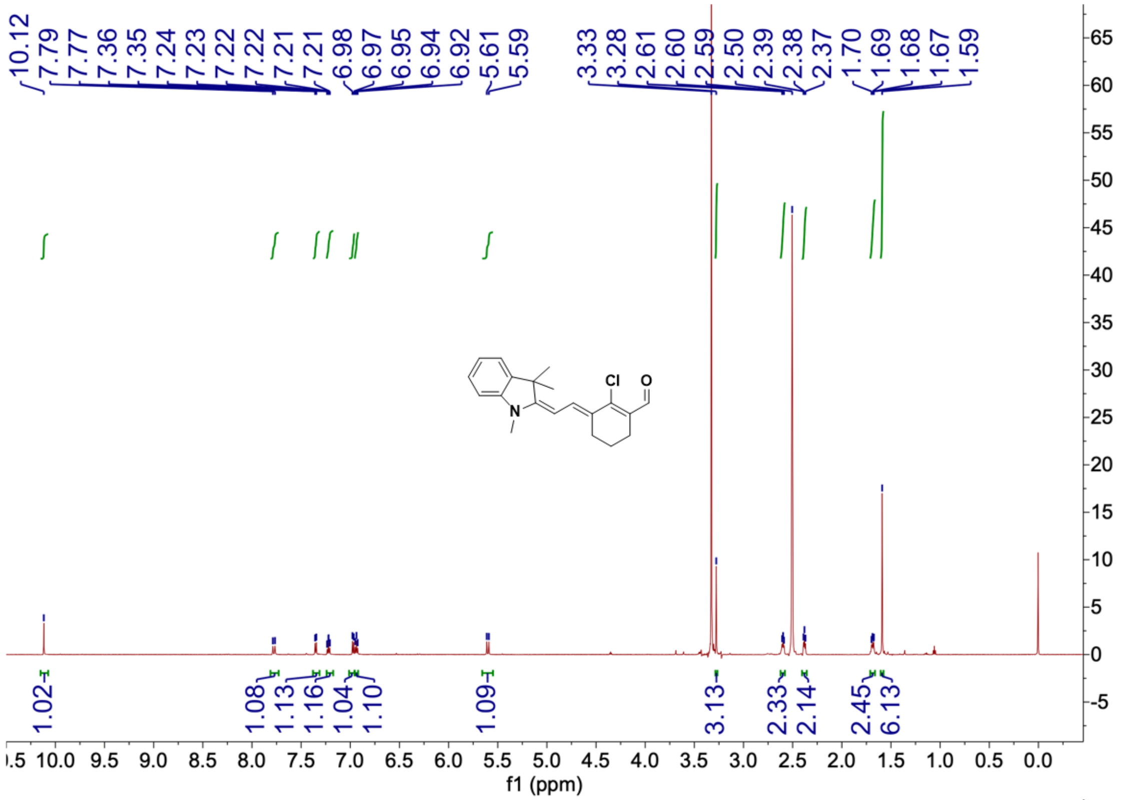
10.12
7.79
7.77
7.36
7.35
7.24
7.23
7.22
7.22
7.21
7.21
6.98
6.97
6.95
6.94
6.92
5.61
5.59
3.33
3.28
2.61
2.60
2.59
2.50
2.39
2.38
2.37
1.70
1.69
1.68
1.67
1.59
Cl
O
N
1.02
1.08
1.13
1.16
1.04
1.10
1.09
3.13
2.33
2.14
2.45
6.13
f1 (ppm)

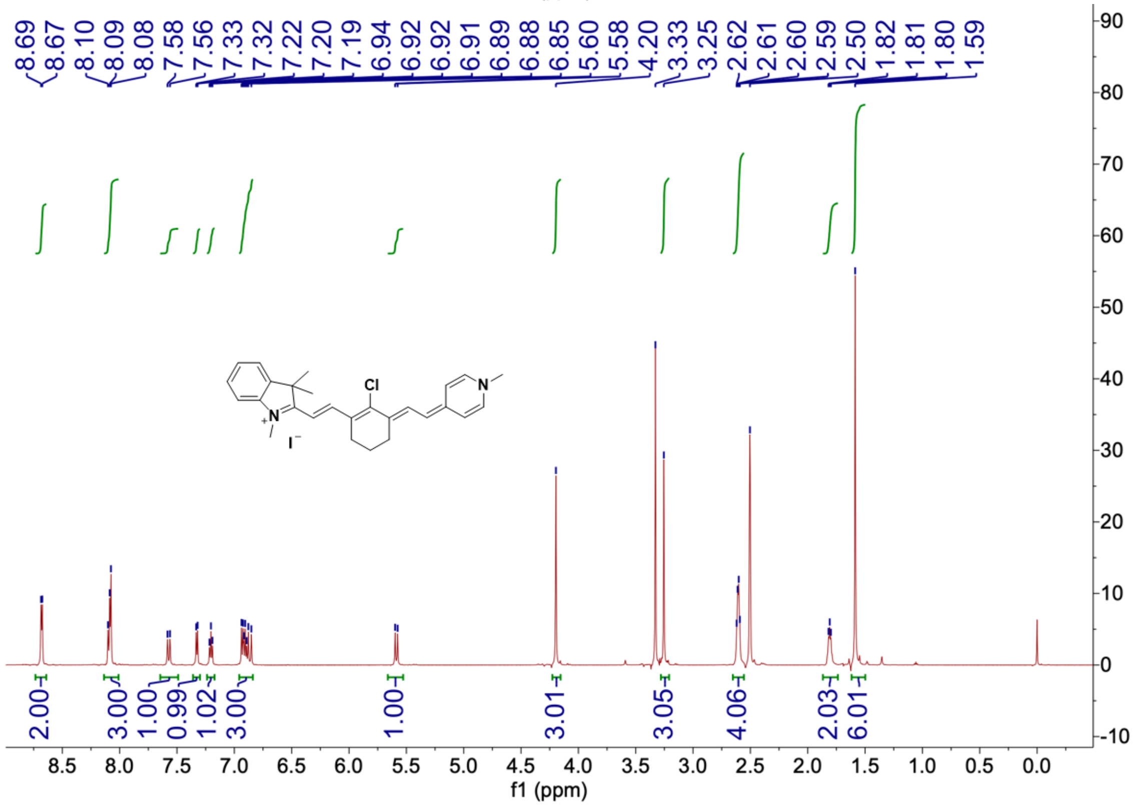
8.69
8.67
8.10
8.09
8.08
7.58
7.56
7.33
7.32
7.22
7.20
7.19
6.94
6.92
6.92
6.91
6.89
6.88
6.85
5.60
5.58
4.20
3.33
3.25
2.62
2.61
2.60
2.59
2.50
1.82
1.81
1.80
1.59
Cl
N
I⁻
2.00
3.00
1.00
0.99
1.02
3.00
1.00
3.01
3.05
4.06
2.03
6.01
f1 (ppm)

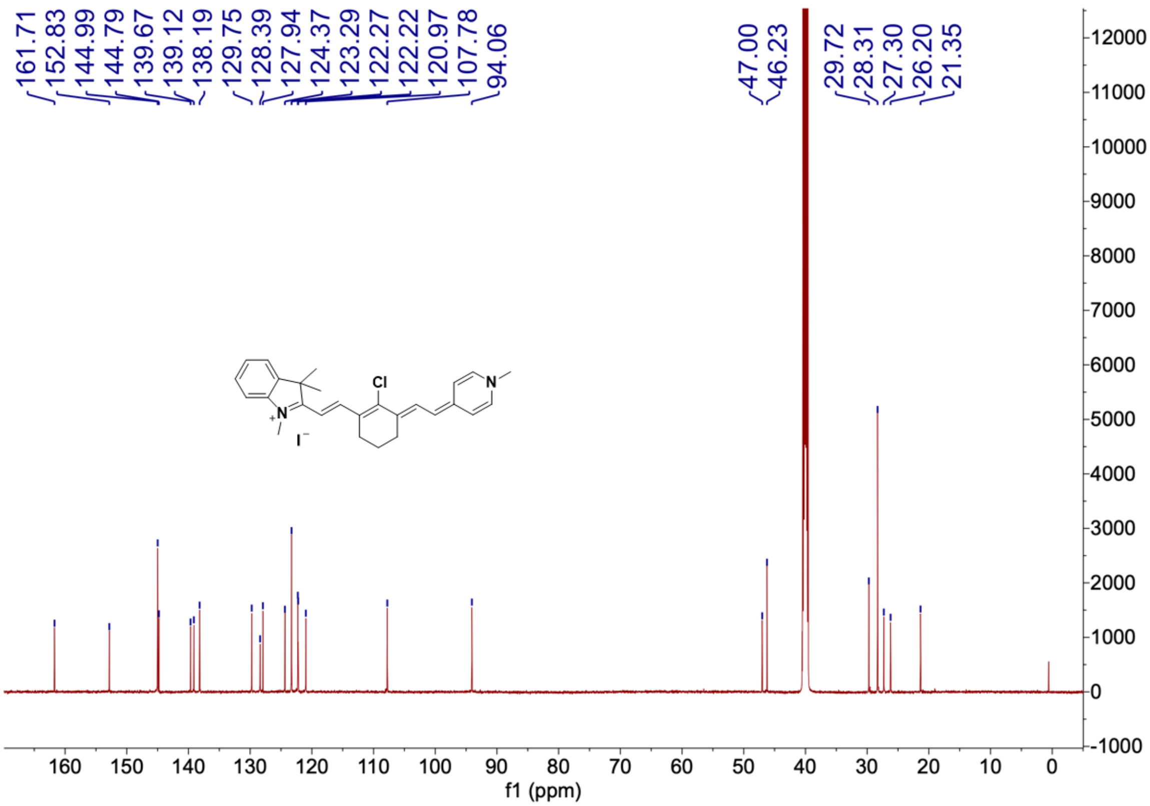
161.71
152.83
144.99
144.79
139.67
139.12
138.19
129.75
128.39
127.94
124.37
123.29
122.27
122.22
120.97
107.78
94.06
47.00
46.23
29.72
28.31
27.30
26.20
21.35
Cl
N
N
I⁻
f1 (ppm)

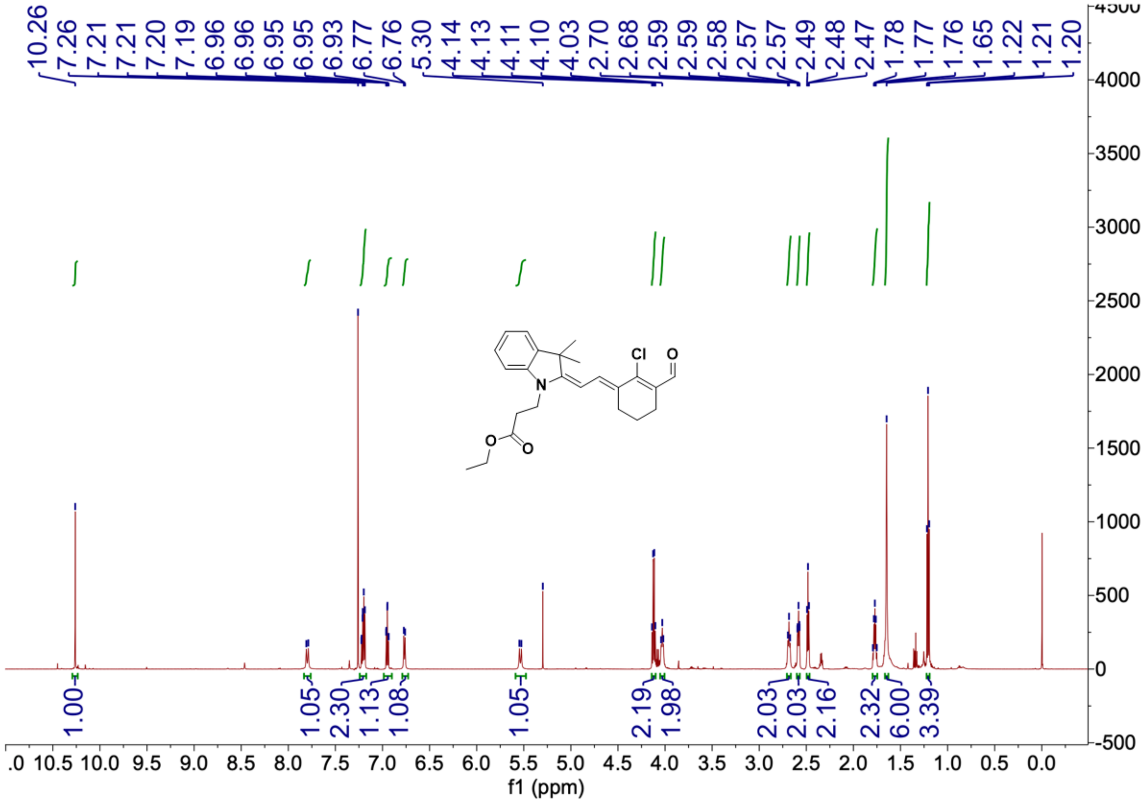
10.26
7.26
7.21
7.21
7.20
7.19
6.96
6.96
6.95
6.95
6.93
6.77
6.76
5.30
4.14
4.13
4.11
4.10
4.03
2.70
2.68
2.59
2.59
2.58
2.57
2.57
2.49
2.48
2.47
1.78
1.77
1.76
1.65
1.22
1.21
1.20
Cl
O
N
O
O
1.00
1.05
2.30
1.13
1.08
1.05
2.19
1.98
2.03
2.03
2.16
2.32
6.00
3.39
f1 (ppm)

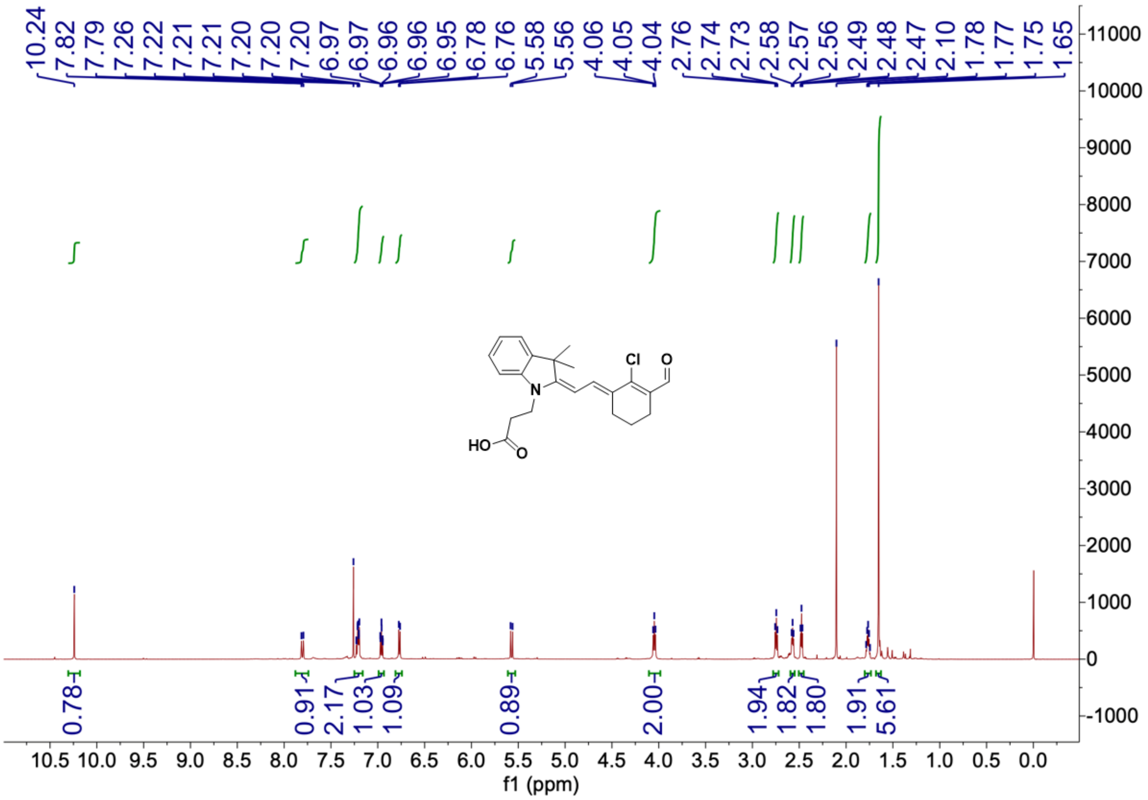

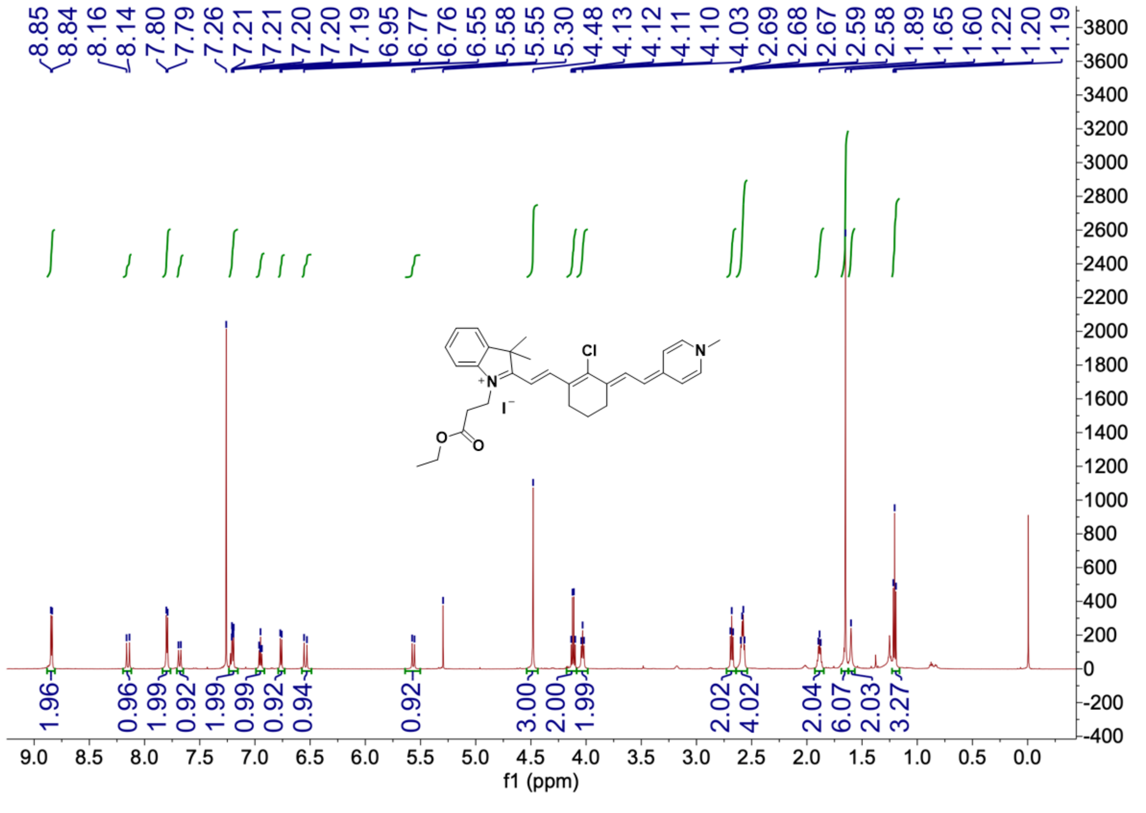

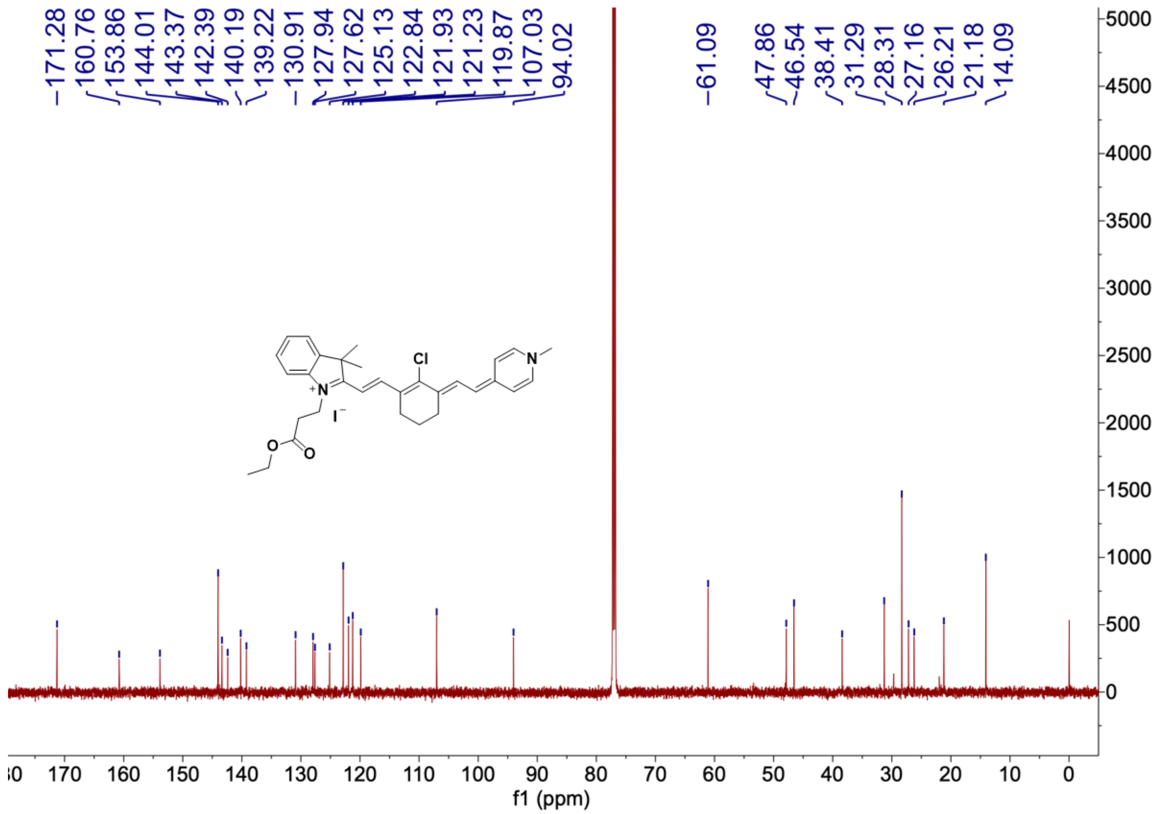
171.28
160.76
153.86
144.01
143.37
142.39
140.19
139.22
130.91
127.94
127.62
125.13
122.84
121.93
121.23
119.87
107.03
94.02
61.09
47.86
46.54
38.41
31.29
28.31
27.16
26.21
21.18
14.09
Cl
N
N
I⁻
O
O
f1 (ppm)

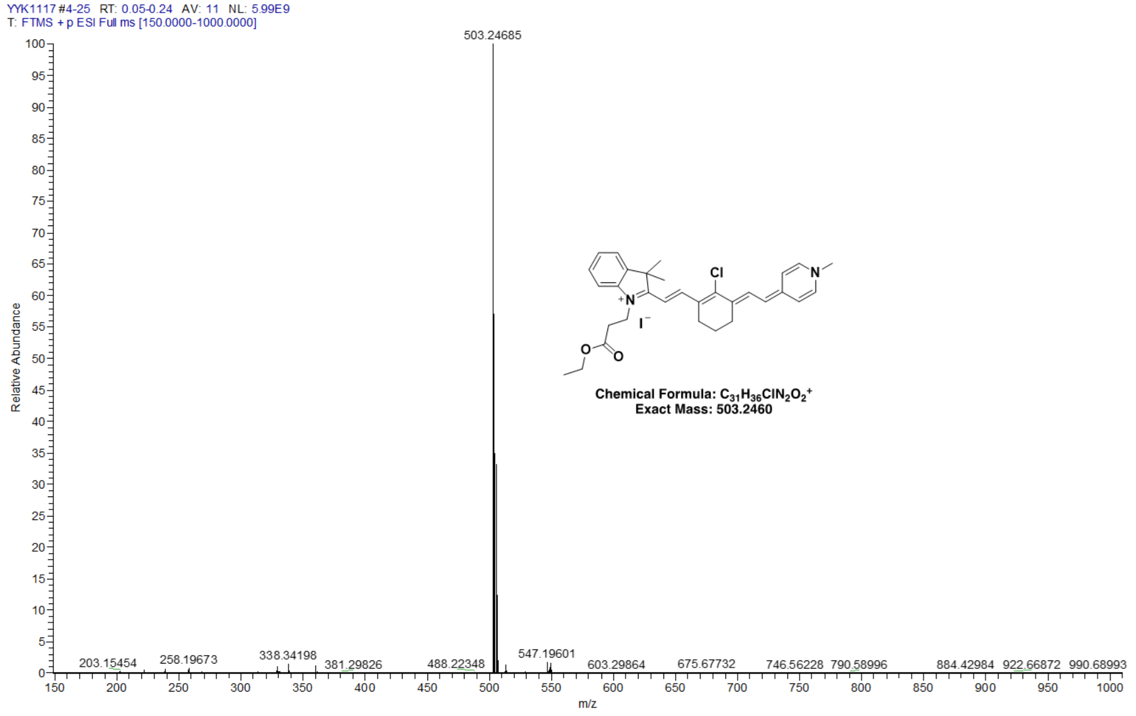
YYK1117 #4-25 RT: 0.05-0.24 AV: 11 NL: 5.99E9
T: FTMS + p ESI Full ms [150.0000-1000.0000]
503.24685
Relative Abundance
203.15454
258.19673
338.34198
381.29826
488.22348
547.19601
603.29864
675.67732
746.56228
790.58996
884.42984
922.66872
990.68993
m/z
Cl
N
N
I⁻
O
O
Chemical Formula: C31H36ClN2O2+
Exact Mass: 503.2460

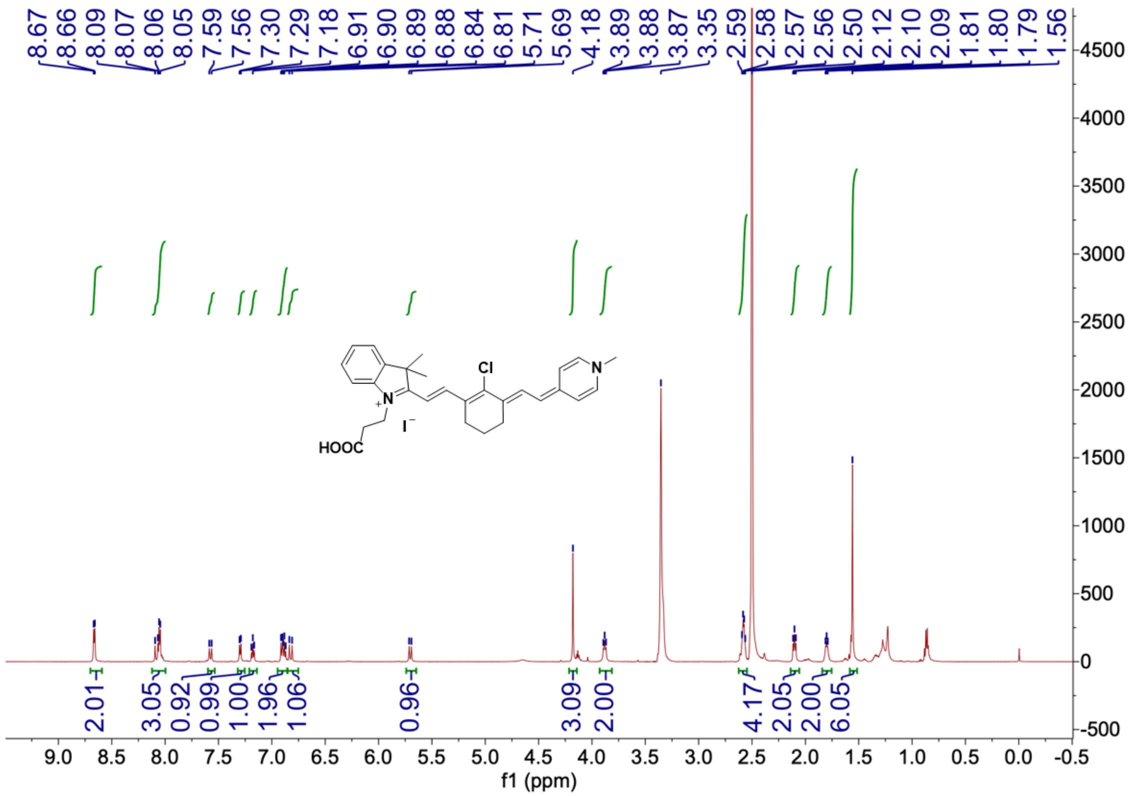
8.67
8.66
8.09
8.07
8.06
8.05
7.59
7.56
7.30
7.29
7.18
6.91
6.90
6.89
6.88
6.84
6.81
5.71
5.69
4.18
3.89
3.88
3.87
3.35
2.59
2.58
2.57
2.56
2.50
2.12
2.10
2.09
1.81
1.80
1.79
1.56
Cl
N
N
HOOC
I−
2.01
3.05
0.92
0.99
1.00
1.96
1.06
0.96
3.09
2.00
4.17
2.05
2.00
6.05
f1 (ppm)

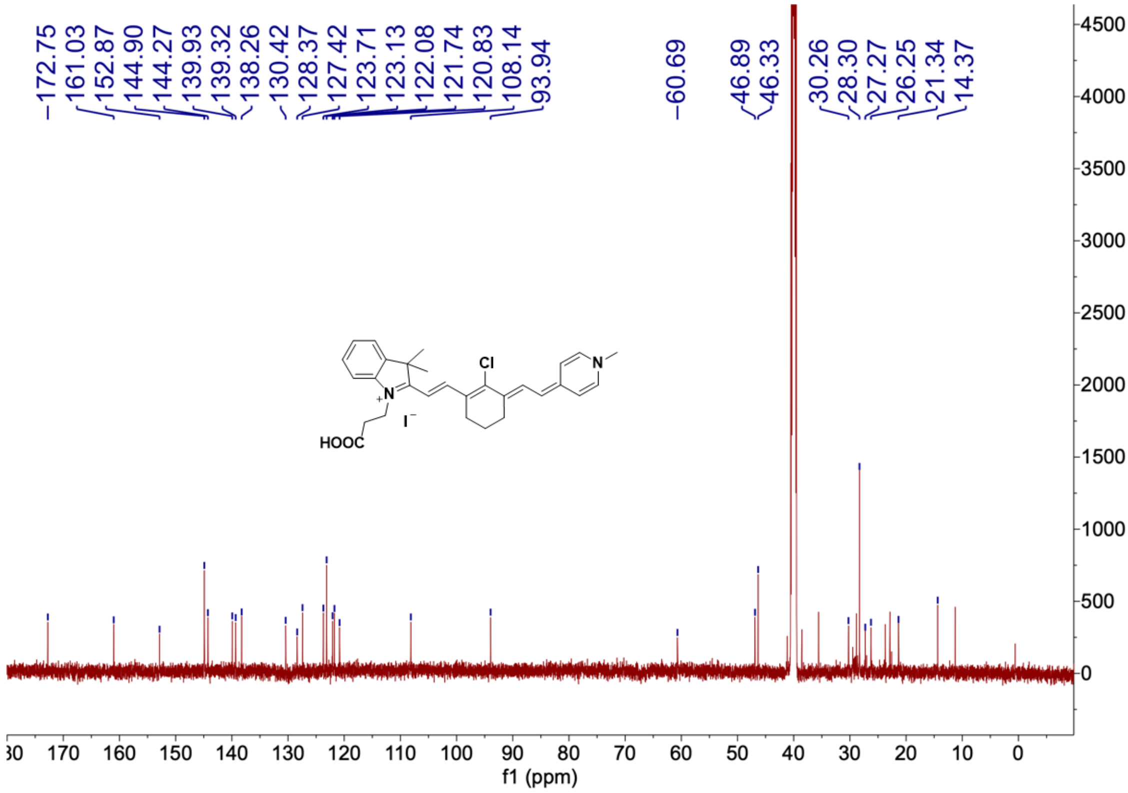
172.75
161.03
152.87
144.90
144.27
139.93
139.32
138.26
130.42
128.37
127.42
123.71
123.13
122.08
121.74
120.83
108.14
93.94
60.69
46.89
46.33
30.26
28.30
27.27
26.25
21.34
14.37
Cl
N
N
HOOC
I−
f1 (ppm)

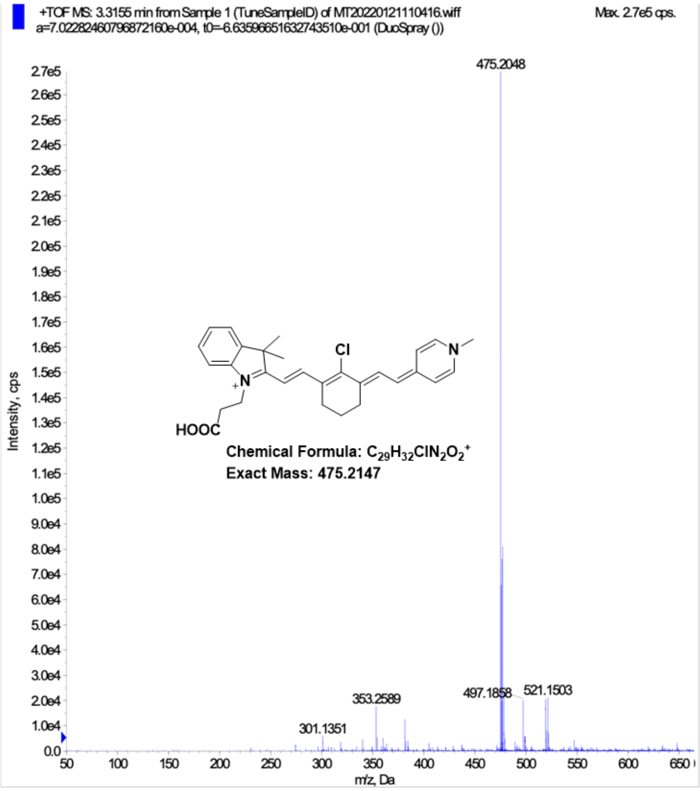
+TOF MS: 3.3155 min from Sample 1 (TuneSampleID) of MT20220121110416.wiff
a=7.02282460796872160e-004, t0=6.63596651632743510e-001 (DuoSpray ())
Max. 2.7e5 cps.
475.2048
353.2589
301.1351
497.1858
521.1503
Intensity, cps
m/z, Da
Cl
N
HOOC
Chemical Formula: $C_{29}H_{32}ClN_2O_2^+$
Exact Mass: 475.2147

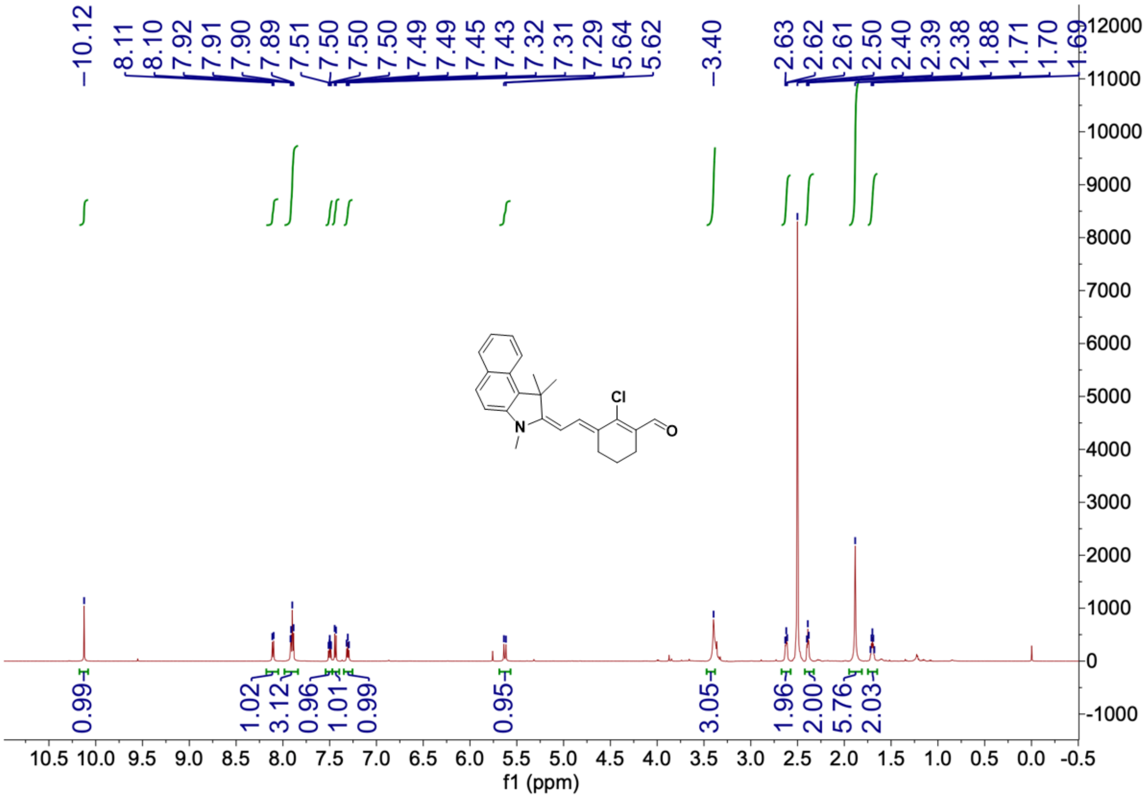
10.12
8.11 8.10 7.92 7.91 7.90 7.89 7.51 7.50 7.50 7.50 7.49 7.49 7.45 7.43 7.32 7.31 7.29 5.64 5.62
3.40
2.63 2.62 2.61 2.50 2.40 2.39 2.38 1.88 1.71 1.70 1.69
0.99 1.02 3.12 0.96 1.01 0.99 0.95 3.05 1.96 2.00 5.76 2.03
Cl
N
O
f1 (ppm)

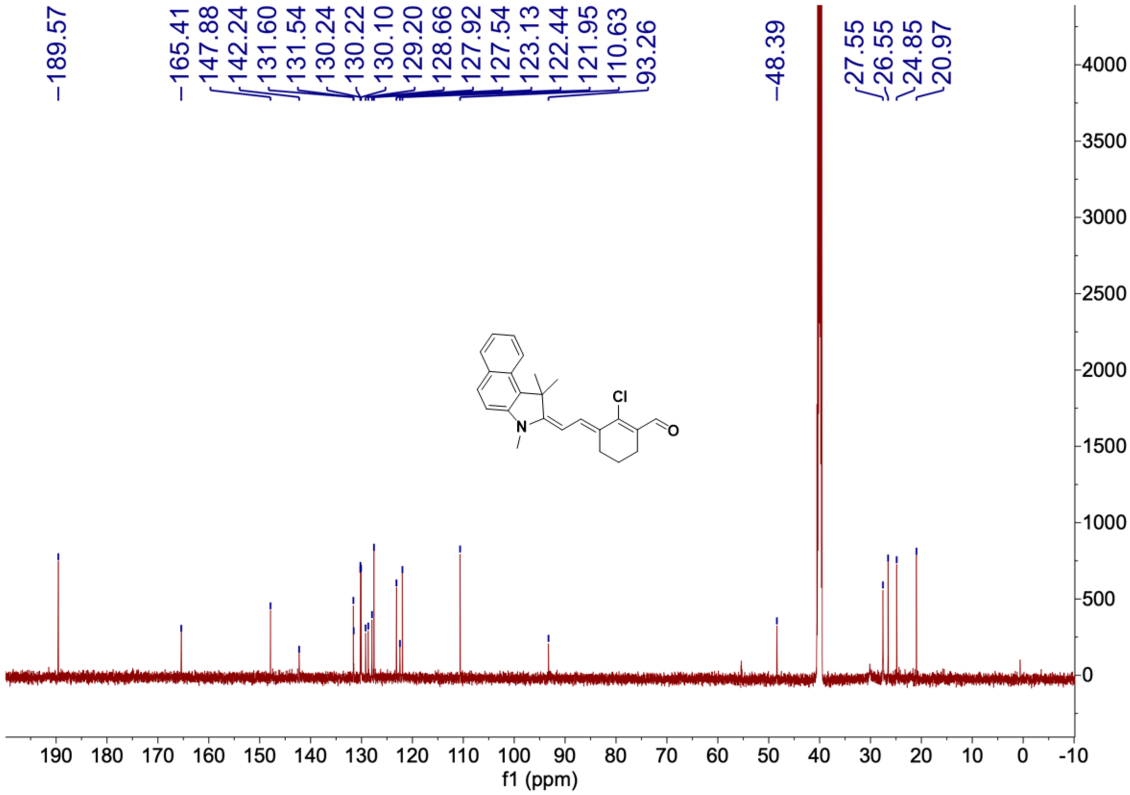

189.57
165.41
147.88
142.24
131.60
131.54
130.24
130.22
130.10
129.20
128.66
127.92
127.54
123.13
122.44
121.95
110.63
93.26
48.39
27.55
26.55
24.85
20.97
Cl
O
N
f1 (ppm)

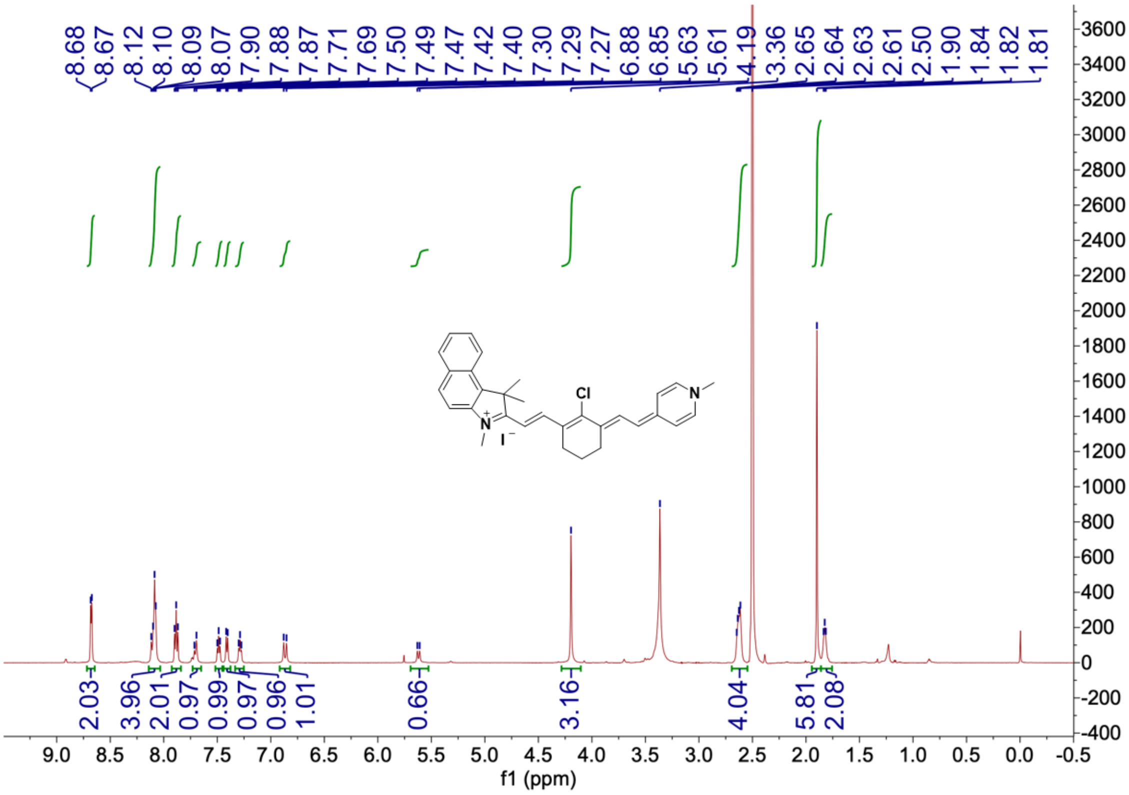

8.68
8.67
8.12
8.10
8.09
8.07
7.90
7.88
7.87
7.71
7.69
7.50
7.49
7.47
7.42
7.40
7.30
7.29
7.27
6.88
6.85
5.63
5.61
4.19
3.36
2.65
2.64
2.63
2.61
2.50
1.90
1.84
1.82
1.81
Cl
N
N
I⁻
2.03
3.96
2.01
0.97
0.99
0.97
0.96
1.01
0.66
3.16
4.04
5.81
2.08
f1 (ppm)

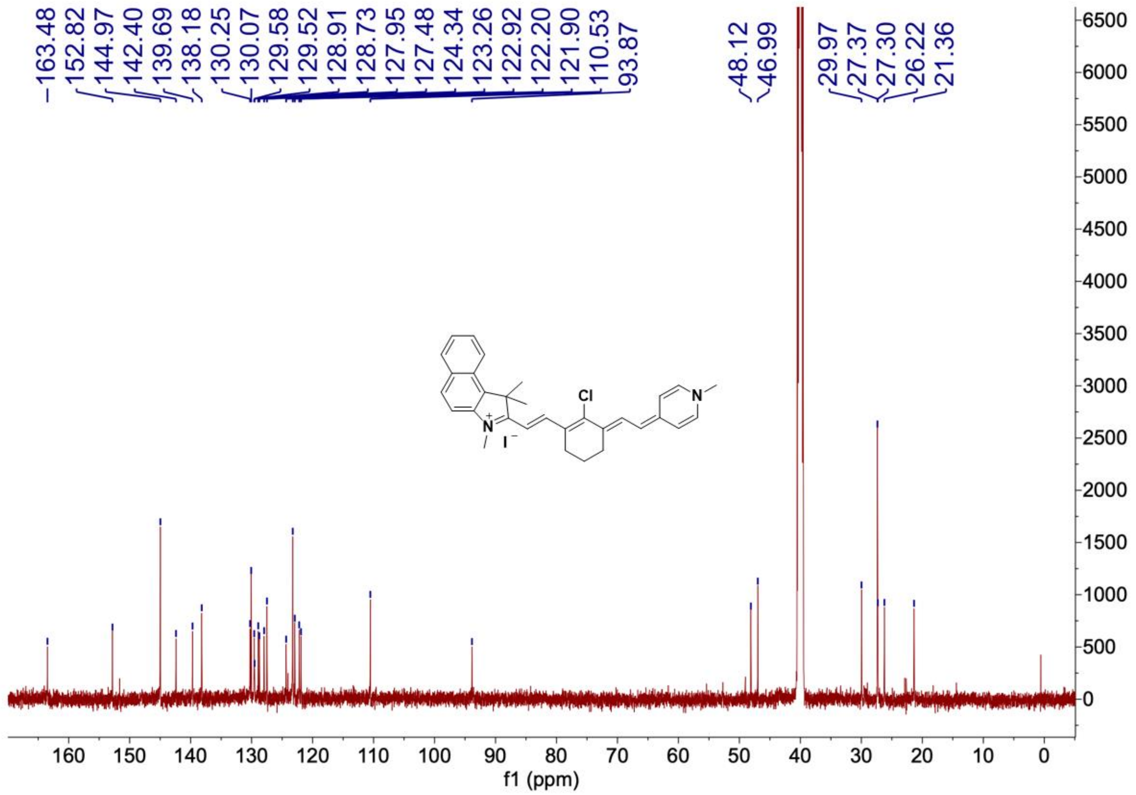

163.48
152.82
144.97
142.40
139.69
138.18
130.25
130.07
129.58
129.52
128.91
128.73
127.95
127.48
124.34
123.26
122.92
122.20
121.90
110.53
93.87
48.12
46.99
29.97
27.37
27.30
26.22
21.36
Cl
N
I⁻
f1 (ppm)

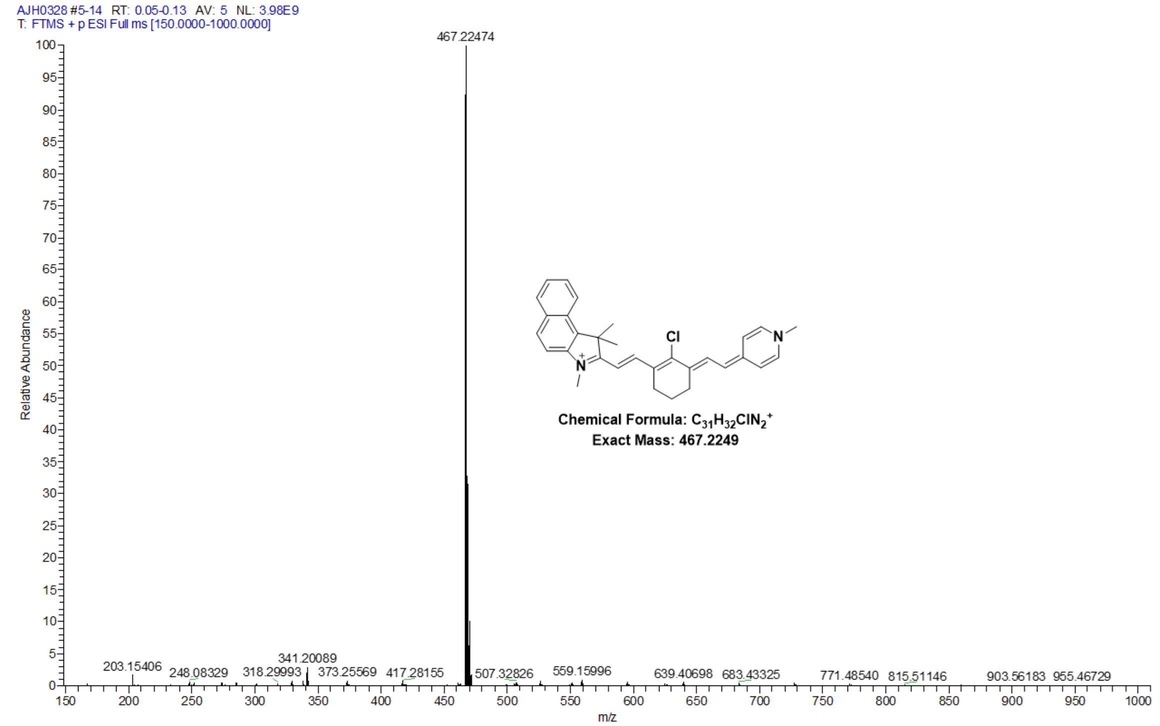

AJH0328 #5-14 RT: 0.05-0.13 AV: 5 NL: 3.98E9
T: FTMS + p ESI Full ms [150.0000-1000.0000]
467.22474
Relative Abundance
203.15406
248.08329
318.29993
341.20089
373.25569
417.28155
507.32826
559.15996
639.40698
683.43325
771.48540
815.51146
903.56183
955.46729
m/z
Cl
N
Chemical Formula: $C_{31}H_{32}ClN_2^+$
Exact Mass: 467.2249

SI References

[1] X. Peng, F. Song, E. Lu, Y. Wang, W. Zhou, J. Fan, Y. Gao, *J. Am. Chem. Soc.* **2005**, *127*, 4170–4171.
